# FN1-associated cavernous endothelial remodeling induced by bisphenol A: relevance to erectile dysfunction and attenuation by baicalin

**DOI:** 10.3389/fnut.2026.1920479

**Published:** 2026-08-13

**Authors:** Xinyao Zhu, Zhen Luo, Qilong Wu, Juan Wang, Chunyang Meng, Yuqi Li, Qingfu Deng

**Affiliations:** 1 Department of Urology, Affiliated Hospital of Southwest Medical University, Luzhou, Sichuan, China; 2 Department of Urology, Beijing Anzhen Nanchong Hospital of Capital Medical University and Nanchong Central Hospital, Nanchong, Sichuan, China; 3 Department of Andrology, Nanjing Drum Tower Hospital, The Affiliated Hospital of Nanjing University Medical School, Nanjing, Jiangsu, China

**Keywords:** baicalin, bisphenol A, diabetic erectile dysfunction, EndMT-like remodeling, endothelial remodeling, erectile dysfunction, FN1, PI3K/AKT/eNOS signaling

## Abstract

**Background:**

Previous studies suggest that bisphenol A (BPA) exposure may impair erectile function, but the underlying cavernous endothelial mechanisms remain unclear. Whether BPA induces endothelial alterations resembling those in diabetic erectile dysfunction (DMED) is unknown.

**Methods:**

BPA targets, DMED-related genes, and self-generated DMED corpus cavernosum transcriptomic data were integrated to identify candidate genes. Protein–protein interaction analysis and machine-learning-assisted feature selection were used to prioritize consensus candidate genes. Human corpus cavernosum single-cell and normal spatial transcriptomic data were analyzed to characterize cell-type-specific expression and endothelial remodeling. Molecular docking and 100-ns molecular dynamics simulations evaluated the predicted structural compatibility of BPA and baicalin with an available FN1 domain. BPA exposure, FN1 knockdown, and delayed baicalin intervention were then evaluated in immortalized rat corpus cavernosum endothelial cells by RT-qPCR, Western blotting, and CCK-8 assays.

**Results:**

Forty-three BPA–DMED-associated candidate genes were identified, with FN1 consistently prioritized by network topology and machine-learning analyses. FN1 was enriched in fibroblasts and a subset of endothelial cells, and FN1-high DMED endothelial cells showed enhanced endothelial-to-mesenchymal transition-like remodeling. Spatial reference analysis showed a positive association between FN1 expression and remodeling scores. *In vitro*, BPA increased FN1, reduced endothelial markers, increased mesenchymal-related markers, and suppressed PI3K/AKT/eNOS phosphorylation, with changes similar in direction to high-glucose stimulation. FN1 knockdown and delayed baicalin intervention partially restored endothelial markers, signaling activity, and cell viability.

**Conclusion:**

FN1-associated endothelial remodeling may represent one component of BPA-related endothelial injury relevant to erectile dysfunction, while baicalin may provide partial protection.

## 1 Introduction

Bisphenol A (BPA) is an industrial chemical widely used in the manufacture of polycarbonate plastics and epoxy resins and is present in food containers, packaging materials, can linings, thermal paper, and a broad range of consumer products (1, 2). BPA can migrate from these materials, resulting in continuous human exposure through food, drinking water, inhalation, and dermal contact. Food-contact materials are considered an important source of BPA exposure in the general population (1, 2). Previous studies have associated BPA exposure with endocrine disruption, metabolic toxicity, vascular dysfunction, and adverse male reproductive effects, including disturbances in insulin signaling, redox homeostasis, inflammatory responses, and endothelial function (3, 4). Epidemiological studies have suggested that higher BPA exposure may be associated with erectile difficulties and impaired overall sexual function (5, 6). Experimental studies have reported marked histological and molecular alterations in rat corpus cavernosum tissue following BPA exposure (7), whereas impaired erectile function has been observed in another BPA-exposed rodent model (8). However, the molecular mechanisms through which BPA may disturb cavernous homeostasis and erectile function remain insufficiently understood.

Erectile dysfunction (ED) is a clinically heterogeneous disorder that may arise from vascular, neurological, endocrine, psychogenic, or iatrogenic causes (9). Consequently, broadly defined ED-related molecular profiles may encompass several biologically distinct pathological processes and may therefore be less suitable for resolving mechanisms associated with a specific environmental exposure. Diabetic erectile dysfunction (DMED), by contrast, has a relatively well-defined metabolic and vascular pathological background characterized by sustained metabolic stress, cavernous endothelial dysfunction, impaired phosphoinositide 3-kinase (PI3K)/protein kinase B (AKT)/endothelial nitric oxide synthase (eNOS) signaling and nitric oxide (NO) bioavailability, extracellular matrix accumulation, and tissue remodeling (10, 11). Endothelial-to-mesenchymal transition-like remodeling (EndMT-like remodeling) may further destabilize the endothelial phenotype and contribute to corpus cavernosum fibrosis (12, 13). Notably, insulin-signaling abnormalities, oxidative stress, chronic inflammation, and endothelial injury associated with BPA exposure overlap substantially with these DMED-related pathological processes. DMED therefore provides a mechanistically defined metabolic–endothelial reference for investigating BPA-related erectile impairment, rather than representing an established disease outcome of BPA exposure.

FN1 is a well-established extracellular matrix glycoprotein involved in matrix assembly, vascular remodeling, and tissue fibrosis. Previous studies have reported increased fibronectin expression in diabetic penile tissue and have used fibronectin as an indicator of corpus cavernosum fibrosis (14). Therefore, the general involvement of FN1 in diabetic fibrosis is not itself novel. However, it remains unclear whether FN1 identifies or contributes to a cell-type-specific cavernous endothelial remodeling state, and whether this process is involved in endothelial injury induced by an environmental toxicant such as BPA. Although an AKT1/FN1-associated fibrogenic program has recently been implicated in BPA-related liver injury (15), the relevance of FN1 to BPA-exposed cavernous endothelial cells and erectile-function-related vascular pathology has not been defined.

Network toxicology integrates chemical targets, disease-associated genes, and molecular interaction data to identify genes and biological pathways that may be perturbed by environmental chemicals (16, 17). Integration with tissue transcriptomics and machine-learning-assisted feature selection may further improve the prioritization of biologically relevant candidate molecules (18, 19). Single-cell transcriptomics can resolve cell-type-specific expression patterns and disease-associated cellular states within the corpus cavernosum, whereas spatial transcriptomics provides complementary anatomical information on the tissue distribution of molecular and cellular features (20, 21). Molecular docking and molecular dynamics simulations can additionally predict candidate docking poses and characterize the simulated dynamic behavior of computationally modeled small-molecule–protein systems. Combining these computational approaches with genetic perturbation and cell-based experiments enables the construction of a multilevel evidence framework spanning candidate prioritization, cellular localization, structural prediction, and functional evaluation.

Based on the overlap between BPA-associated toxicity and DMED-related endothelial pathology, we hypothesized that BPA may converge on a DMED-relevant cavernous endothelial remodeling program. Rather than selecting a specific mediator *a priori*, we integrated predicted BPA targets, DMED-related genes, self-generated and public transcriptomic data, PPI-network topology, and machine-learning-assisted feature selection to identify consensus candidate molecules. FN1 emerged as the only candidate jointly prioritized by three network-topology algorithms and two machine-learning approaches and was therefore selected for subsequent cell-type-resolved and functional analyses. Human corpus cavernosum single-cell transcriptomic data and normal spatial transcriptomic reference data were then used to characterize the cellular distribution of FN1 and its association with endothelial remodeling. Finally, molecular simulations, FN1 knockdown, and delayed baicalin intervention were used to evaluate the potential involvement of FN1 in BPA-associated endothelial abnormalities.

## 2 Materials and methods

### 2.1 Chemical information and toxicity profiling of BPA

The two-dimensional and three-dimensional chemical structures, Chemical Abstracts Service (CAS) registry number, molecular formula, molecular weight, and canonical simplified molecular-input line-entry system (SMILES) of bisphenol A (BPA) were obtained from PubChem (https://pubchem.ncbi.nlm.nih.gov/). The canonical SMILES of BPA was submitted to the ProTox-3.0 web server (https://tox.charite.de/protox3/) to predict its potential toxicity profile, including acute toxicity, organ toxicity, toxicological endpoints, toxicity-associated molecular targets, and related toxicological pathways. The predicted probabilities and confidence scores generated by the platform were used to construct a radar plot of the toxicity characteristics of BPA. The chemical structure and SMILES information of BPA were also used for subsequent target prediction, molecular docking, and molecular dynamics simulations.

### 2.2 Prediction of potential BPA targets

Potential molecular targets of BPA were collected from the Comparative Toxicogenomics Database (CTD; https://ctdbase.org/), SwissTargetPrediction (https://www.swisstargetprediction.ch/), and the Similarity Ensemble Approach (SEA; https://sea.bkslab.org/). In CTD, “bisphenol A” was used as the search term, and genes with an interaction count >15 were retained to improve the reliability of target screening. The SMILES of BPA was separately submitted to SwissTargetPrediction and SEA, with the species restricted to Homo sapiens. Targets obtained from the three databases were converted to official gene symbols, combined, and deduplicated to generate the final BPA target set.

### 2.3 Collection of DMED-related genes and transcriptomic analysis

DMED-related genes were retrieved from GeneCards using “diabetic erectile dysfunction,” and genes with relevance scores >15 were retained. The self-generated rat corpus cavernosum transcriptomic dataset included five control and five DMED samples. The type 2 diabetic rat model was established as previously described (22). For corpus cavernosum tissue collection, rats were anesthetized using a vaporizer-equipped small-animal anesthesia system with isoflurane delivered in oxygen. Anesthesia was induced with 3%−5% isoflurane and maintained with 1.5%−2.0% isoflurane at an oxygen flow rate of 1–2 L/min. Following completion of the experimental procedures, the rats were euthanized by isoflurane overdose. The isoflurane concentration was increased to 5% and maintained until spontaneous respiration ceased, after which exposure was continued for at least 1 min. Death was confirmed by the absence of spontaneous respiration and heartbeat before the corpus cavernosum tissues were collected. The rat corpus cavernosum transcriptomic data used in this study were generated from animal experiments conducted in accordance with institutional guidelines for the care and use of laboratory animals. The animal experimental protocol was approved by the Animal Ethics Committee of Southwest Medical University (approval no. swmu20250608). Differentially expressed genes were identified using limma with |log2 fold change| >1 and *P* < 0.05. Rat genes were converted to human orthologs, and BPA targets, DMED-related genes, and differentially expressed genes were intersected to obtain candidate genes.

### 2.4 Functional enrichment analysis

Gene enrichment analysis identifies biological pathways or functions that are over-represented in a given set of genes, thereby providing insights into the underlying biological processes and potential molecular mechanisms (23–25). Gene Ontology (GO) and Kyoto Encyclopedia of Genes and Genomes (KEGG) enrichment analyses of the BPA–DMED-associated candidate genes were performed using the clusterProfiler and org.Hs.eg.db R packages. GO analysis included the biological process, cellular component, and molecular function categories, whereas the species for KEGG analysis was set to Homo sapiens. The Benjamini–Hochberg method was used to correct for multiple testing, and an adjusted *P* value < 0.05 was considered statistically significant.

### 2.5 Construction of the PPI network and hub-gene prioritization

The BPA–DMED-associated candidate genes were submitted to the Search Tool for the Retrieval of Interacting Genes/Proteins database (STRING; https://string-db.org/) to construct a protein–protein interaction (PPI) network. The species was restricted to Homo sapiens, and the minimum interaction confidence score was set to 0.400. After disconnected nodes were removed, the PPI network was imported into Cytoscape (https://cytoscape.org/) for visualization and topological analysis.

The maximal clique centrality (MCC), maximum neighborhood component (MNC), and Degree algorithms implemented in the cytoHubba plugin were used to rank the nodes, and the top 10 genes identified by each algorithm were retained. The topological screening results were subsequently integrated with the machine-learning-assisted feature-selection results to prioritize candidate genes for downstream analyses.

### 2.6 Integration of the self-generated DMED dataset with GSE2457 and machine-learning-assisted feature selection

The public transcriptomic dataset GSE2457 was downloaded from the Gene Expression Omnibus (GEO; https://www.ncbi.nlm.nih.gov/geo/). Five control samples (GSM46500, GSM46503, GSM46508, GSM46512, and GSM46516) and five diabetes-related samples (GSM46518, GSM46519, GSM46520, GSM46521, and GSM46522) were included.

After gene annotation, ortholog conversion, and matching of shared genes, the self-generated DMED transcriptomic dataset was merged with GSE2457. Batch effects between the two datasets were corrected using the ComBat function in the sva R package. Expression-distribution boxplots, hierarchical clustering, and principal component analysis (PCA) were used to evaluate the effectiveness of batch correction.

The input features for machine-learning-assisted screening were restricted to genes shared among the BPA target set, DMED-related genes, and DEGs from the self-generated DMED dataset. Least absolute shrinkage and selection operator (LASSO) regression was performed using the glmnet R package. A binomial model was fitted with alpha set to 1. Ten-fold cross-validation based on binomial deviance was used to determine the optimal penalty parameter, and genes with non-zero coefficients at lambda.min were retained as the LASSO-selected features.

Support vector machine–recursive feature elimination (SVM-RFE) was performed using the e1071 R package and a linear kernel. Gene-expression values were standardized before analysis. Ten-fold cross-validation was used to evaluate classification error across different feature-set sizes, and the feature set corresponding to the lowest mean cross-validation error was selected. The MCC, MNC, Degree, LASSO, and SVM-RFE results were subsequently integrated to identify jointly prioritized BPA–DMED candidate genes.

### 2.7 Single-cell transcriptomic data acquisition and quality control

Single-cell RNA-sequencing data were obtained from GEO dataset GSE206528. Two normal reference samples (GSM6255907, Normal_1; and GSM6255908, Normal_2) and two diabetic erectile dysfunction samples (GSM6255913, Diabetes_1; and GSM6255914, Diabetes_2) were included to obtain comparable cell numbers between the normal and disease groups. The normal reference samples were derived from non-tumorous tissue adjacent to tumors in patients with preserved erectile function, whereas the diabetic erectile dysfunction samples were obtained from biopsies collected during penile prosthesis implantation.

Quality control and downstream analyses were performed using the Seurat R package. The number of unique molecular identifiers (UMIs), the number of detected genes, the percentage of mitochondrial transcripts, and the percentage of ribosomal transcripts were calculated for each cell. Cells were retained if they met all of the following criteria: nCount_RNA ≥ 1,000, 200 ≤ nFeature_RNA ≤ 10,000, percent.mt ≤ 20%, and percent.rb ≤ 20%. Cells that did not meet these criteria were excluded as low-quality or potentially abnormal cells.

### 2.8 Single-cell data normalization, batch correction, dimensionality reduction, clustering, and cell annotation

After quality control, the single-cell expression matrices were normalized using the LogNormalize method with a scale factor of 10,000. Highly variable genes were identified, followed by data scaling and PCA. Harmony was used to correct sample-level batch effects. A nearest-neighbor graph was constructed in the Harmony-corrected low-dimensional space, and graph-based clustering was performed using the FindClusters function. Uniform manifold approximation and projection (UMAP) was used to visualize the cellular populations.

Cell types were annotated by integrating cluster-specific expression patterns with canonical marker genes. The candidate lineages assessed included endothelial cells (PECAM1, VWF, CDH5, and CLDN5), fibroblasts (LUM, COL1A1, PDGFRA, and DCN), pericytes (RGS5, KCNJ8, PDGFRB, and CSPG4), smooth muscle cells (ACTA2, MYH11, TAGLN, and MYLK), Schwann/neural-related cells (S100B, MPZ, and PLP1), myeloid cells (CD163, CD68, AIF1, and CSF1R), and T cells (CD3D, CD3E, CD3G, and CD2). The final annotated cell populations comprised endothelial cells, fibroblasts, smooth muscle cells, macrophages, and T cells.

### 2.9 Pseudotemporal analysis of DMED cells

To explore potential transcriptional-state trajectories in the DMED corpus cavernosum, all annotated cells from the DMED samples were extracted and subjected to pseudotemporal analysis using Monocle. Ordering genes were selected according to gene-expression variability and cell-population marker profiles, followed by dimensionality reduction and trajectory construction. The root state was selected according to the inferred trajectory structure and the distribution of the major cell types. Cells were visualized according to pseudotime, cell type, and branch state.

This analysis was used only to describe potential relationships among transcriptional states in DMED tissue and was not interpreted as evidence of a true lineage transition or direct conversion of endothelial cells into fibroblasts.

### 2.10 FN1 expression, FN1-high/low grouping, and assessment of EndMT-like remodeling

FN1 expression was examined across the annotated cell types and disease groups. Endothelial cells from the DMED samples were subsequently extracted and reprocessed to characterize their transcriptional heterogeneity.

DMED endothelial cells were divided into FN1-high and FN1-low groups according to the median FN1 expression for subsequent pathway-activity and endothelial-to-mesenchymal transition-like (EndMT-like) remodeling analyses. The Hallmark epithelial–mesenchymal transition gene set (HALLMARK_EPITHELIAL_MESENCHYMAL_TRANSITION) was obtained from the Molecular Signatures Database (MSigDB; version 2024.1.Hs) and was used as a surrogate transcriptional signature of EndMT-like mesenchymal and extracellular-matrix remodeling in annotated endothelial cells. FN1 was excluded from the gene set before score calculation to avoid a direct circular association between FN1 expression and the resulting score. The complete gene list is provided in Supplementary Table S1. The AddModuleScore function in Seurat was used to calculate an EndMT-like remodeling score for each cell by subtracting the average expression of expression-matched control genes from the average expression of the signature genes. The signature genes were not assigned gene-specific weights, and the score was analyzed as a continuous variable without applying a predefined threshold to classify cells as EndMT-positive or EndMT-negative. Because the Hallmark EMT gene set is not endothelial-specific, the resulting score was interpreted as a relative measure of EndMT-like remodeling rather than as definitive evidence of complete EndMT or endothelial lineage transition.

### 2.11 GSVA and virtual FN1 perturbation analysis

The human Hallmark gene sets were obtained from the Molecular Signatures Database (MSigDB; https://www.gsea-msigdb.org/gsea/msigdb/) using the file h.all.v2024.1.Hs.symbols.gmt (version 2024.1.Hs). Gene set variation analysis (GSVA) was used to compare relative pathway activity between FN1-high and FN1-low DMED endothelial cells and to characterize differences in inflammation-, stress-, metabolism-, and remodeling-related biological programs.

Virtual FN1 perturbation was performed in DMED endothelial cells using scTenifoldKnk to predict transcriptional response genes potentially affected by FN1 perturbation. Genes showing relatively strong predicted perturbation responses were subjected to GO and KEGG enrichment analyses to characterize functional networks potentially associated with endothelial remodeling. These results were interpreted as computational predictions rather than evidence from an actual genetic knockout experiment.

### 2.12 Spatial transcriptomic processing and single-cell reference mapping

Spatial transcriptomic data were obtained from GEO dataset GSE261085, which contains one human corpus cavernosum sample with normal erectile function. The spatial section was derived from a cross-section of human corpus cavernosum tissue and was selected to include representative anatomical regions, including the cavernous artery, septum pectiniforme, tunica albuginea, and most of the cavernosal trabecular region. The spatial dataset was therefore used as a normal anatomical reference rather than as a DMED spatial dataset. The expression matrix was imported into Seurat, and the two-dimensional coordinates of each spatial spot were incorporated into the metadata. The spatial data were normalized, screened for variable genes, scaled, and subjected to PCA, nearest-neighbor graph construction, clustering, and UMAP. Spatial plots based on spot coordinates were generated to visualize spatial clusters, FN1 expression, EndMT-like remodeling scores, and predicted cell-type distributions.

The previously annotated single-cell transcriptomic dataset was used as the reference to infer cellular features within the spatial spots. Cell-type labels were transferred using the anchor-based label-transfer framework implemented in Seurat. The single-cell reference and spatial datasets were separately processed using SCTransform normalization and PCA. Cross-dataset anchors were identified using FindTransferAnchors, and TransferData was used to assign a predicted cell type and prediction score to each spatial spot. These annotations were interpreted as the predominant cell-type signature within each spot rather than as definitive single-cell identities. Compatibility between the datasets was supported by their common human corpus cavernosum origin, shared gene features, and anchor-based integration after separate SCTransform normalization and dimensionality reduction. However, because the spatial dataset was derived from normal tissue whereas the single-cell reference included both normal and DMED samples, label transfer was restricted to broad cell-type signatures and was not used to assign DMED-specific cellular states to spatial spots.

The same Hallmark EMT-derived signature used in the single-cell analysis was applied to the spatial dataset to calculate a spot-level EndMT-like remodeling score using AddModuleScore. FN1 expression and EndMT-like remodeling scores were extracted for all spots, and their spot-level association was assessed using Spearman correlation analysis. Spots were divided into FN1-high and FN1-low groups according to the median FN1 expression, and EndMT-like remodeling scores were compared between the two groups using the Wilcoxon rank-sum test. Because all spots were derived from a single normal human sample, these analyses were considered descriptive assessments of the spatial organization of FN1-associated remodeling features.

### 2.13 Collection of candidate phytochemicals and batch blind docking

Candidate traditional medicine- and plant-derived compounds associated with FN1 were collected from HERB 2.0 (http://herb.ac.cn/) using FN1 as the target query. Compound names were standardized and deduplicated, and compounds without an unambiguous chemical structure were excluded. Three-dimensional compound structures were obtained from PubChem, energy-minimized, and converted into formats suitable for molecular docking.

Information on human FN1 was obtained from UniProt (https://www.uniprot.org/; entry P02751). Because an experimentally determined full-length structure of human FN1 was not available, the experimentally resolved solution NMR structure of an isolated FN1 domain was retrieved from the RCSB Protein Data Bank and used as a local structural model for exploratory molecular docking and molecular dynamics simulations (PDB ID: 2EC3). The 2EC3 structure represents only a 68-residue fragment of the multidomain FN1 protein rather than the full-length protein. Its selection was primarily based on the availability of an experimentally resolved atomic structure and computational feasibility, rather than on prior evidence that this domain contains a physiologically established BPA- or baicalin-binding site. Protein preparation included removal of water molecules and non-essential small molecules, addition of hydrogen atoms, and charge assignment.

Batch blind docking was performed using AutoDock Vina. The docking grid encompassed the entire 2EC3 domain, and no predefined or experimentally validated small-molecule-binding pocket was specified. Each ligand was therefore allowed to explore candidate docking regions across the available domain surface. Candidate compounds were ranked according to their lowest predicted binding energies. Baicalin was selected for subsequent molecular dynamics simulations and cell-based experiments because of its relatively favorable predicted binding energy and potential endothelial-protective relevance.

### 2.14 Molecular docking of BPA–FN1 and baicalin–FN1

The three-dimensional structures of BPA and baicalin were obtained from PubChem. AutoDock Vina was used to perform blind docking of BPA and baicalin across the entire available 2EC3 domain. The pose with the lowest predicted binding energy and a structurally plausible conformation was selected for subsequent visualization and molecular dynamics simulations. Because the docking region was computationally identified rather than selected from an experimentally validated ligand-binding site, the predicted pose was interpreted only as a potential local interaction conformation.

The selected predicted docking poses were imported into Discovery Studio for three-dimensional and two-dimensional visualization of predicted hydrogen-bonding, hydrophobic, π-related, and van der Waals contacts.

### 2.15 Molecular dynamics simulations and MM/GBSA binding free-energy calculations

All-atom molecular dynamics simulations of the BPA–FN1 and baicalin–FN1 complexes were performed using GROMACS. The AMBER99SB force field was used for the protein, and the transferable intermolecular potential with three points (TIP3P) model was used for water molecules. Each protein–ligand complex was placed in a cubic water box, and sodium and chloride ions were added to neutralize the system. Energy minimization was followed by canonical-ensemble equilibration at constant particle number, volume, and temperature (NVT) and isothermal–isobaric equilibration at constant particle number, pressure, and temperature (NPT). A 100-ns production simulation was subsequently conducted at 310 K and 1 bar.

After completion of the simulations, the trajectories were evaluated using the root mean square deviation (RMSD) of the complex, root mean square fluctuation (RMSF) of protein residues, radius of gyration (Rg), solvent-accessible surface area (SASA), protein–ligand hydrogen-bond number, and free-energy landscape.

Binding free energies were estimated from the molecular dynamics trajectories using gmx_MMPBSA. Molecular mechanics/generalized Born surface area (MM/GBSA) calculations were performed using the generalized Born solvent model with igb=5 and an ionic strength of 0.15 M. Frames 500–1,000 were sampled at intervals of 10 frames, yielding 51 snapshots for free-energy calculations. The estimated binding free energy was decomposed into van der Waals, electrostatic, polar solvation, and non-polar solvation components. Entropic contributions were not included; therefore, the calculated values were used to compare the relative energetic favorability of the complexes rather than as absolute experimental binding free energies.

### 2.16 Cell culture, BPA treatment, FN1 knockdown, and delayed baicalin intervention

Immortalized rat corpus cavernosum endothelial cells were obtained from iCell Bioscience (https://www.icellbioscience.com/; catalog no. iCell-0106a). Cells were cultured in Dulbecco's modified Eagle medium (DMEM) supplemented with 10% fetal bovine serum and 1% penicillin–streptomycin at 37 °C in a humidified atmosphere containing 5% CO_2_.

BPA was purchased from Macklin (catalog no. B802576), and baicalin was purchased from Macklin (catalog no. B802696). BPA and baicalin stock solutions were prepared in dimethyl sulfoxide (DMSO). Within each experiment, the vehicle volume was matched among the corresponding treatment groups, and the final DMSO concentration was maintained below 0.1% (v/v). A control+DMSO group was included to evaluate the effect of the vehicle alone. For the delayed-intervention experiment, groups not receiving baicalin were administered an equal volume of DMSO at the corresponding 12-h time point.

The BPA concentration range used in this study was selected with reference to a previous vascular toxicity study, in which human umbilical vein endothelial cells (HUVECs) were exposed to 50–800 μM BPA for 24 h, and 250 μM BPA was subsequently selected for downstream experiments (26). Because the sensitivity to BPA may differ among endothelial cell types, preliminary concentration-screening experiments were performed in corpus cavernosum endothelial cells. Preliminary CCK-8 screening indicated that exposure to 150 μM BPA for 24 h resulted in a mean decrease of less than 25% in cell viability. In parallel, cells were exposed to 0, 50, 100, or 150 μM BPA for 24 h, followed by measurement of FN1 mRNA expression using reverse-transcription quantitative polymerase chain reaction (RT-qPCR). Based on the concentration-dependent increase in FN1 expression and the preliminary CCK-8 results, 150 μM BPA for 24 h was selected as the treatment condition for subsequent experiments.

The first experimental phase compared endothelial alterations induced by high glucose (HG) and BPA. Cells were assigned to the control, control+DMSO, HG, and BPA groups. The HG group was treated with 30 mM glucose for 48 h and served as a DMED-like endothelial injury reference, whereas the BPA group was treated with 150 μM BPA for 24 h. This experiment was designed to compare the direction of molecular alterations induced by HG and BPA and was not used to determine whether BPA aggravated HG-induced injury.

FN1-targeting small interfering RNAs (siFN1) and a negative-control small interfering RNA (siNC) were synthesized by PPL. Three candidate siFN1 sequences were individually transfected into cells, and FN1 knockdown efficiency was assessed by RT-qPCR 48 h after transfection. The siFN1 sequence producing the greatest reduction in FN1 mRNA expression was selected for subsequent experiments.

The second experimental phase included the siNC+BPA, siNC+BPA+baicalin, siFN1+BPA, and siFN1+BPA+baicalin groups. At 48 h after small interfering RNA (siRNA) transfection, all groups were exposed to 150 μM BPA. After 12 h of BPA exposure, 15 μM baicalin was added to the designated intervention groups without removing BPA, and the cells were co-incubated with BPA and baicalin for a further 12 h. The corresponding non-baicalin groups received an equal volume of vehicle at the same time point. The total duration of BPA exposure was 24 h, after which the cells were collected for subsequent analyses. This delayed-intervention design was intended to evaluate the protective effect of baicalin after BPA exposure had already been initiated, rather than under a prophylactic pretreatment condition. Because earlier or later baicalin-administration time points were not compared, the present study could not determine whether the timing of baicalin addition influenced the magnitude of rescue or identify an optimal intervention window.

The 150 μM BPA concentration was used as a short-term *in vitro* exposure condition for mechanistic exploration and was not intended to reproduce circulating BPA concentrations associated with typical human environmental exposure. Similarly, the 15 μM baicalin concentration was used as an *in vitro* pharmacological intervention condition and was not intended to represent a clinically established circulating concentration.

### 2.17 CCK-8 cell-viability assay

The Cell Counting Kit-8 (CCK-8) assay was used to evaluate the effects of FN1 knockdown and delayed baicalin intervention on BPA-associated reductions in cell viability. Cells were seeded into 96-well plates at approximately 4 × 10^3^ cells per well in 100 μL of complete culture medium and were subsequently subjected to siRNA transfection and drug treatment according to the experimental design.

The experimental groups were control, control+DMSO, siNC+BPA, siNC+BPA+baicalin, siFN1+BPA, and siFN1+BPA+baicalin. At 48 h after siRNA transfection, 150 μM BPA was added. After 12 h of BPA exposure, 15 μM baicalin was added to the designated groups, and the cells were co-incubated with BPA and baicalin for an additional 12 h. The control+DMSO group received an equivalent volume of DMSO.

After treatment, 10 μL of CCK-8 reagent was added to each well, followed by incubation at 37 °C for 1.5 h. Absorbance was measured at 450 nm using a microplate reader. Blank wells containing culture medium and CCK-8 reagent but no cells were used for background correction. Cell viability was calculated as follows:


$$\begin{array}{r} \text{Cell viability }(\%)\text{ = }[({\text{OD}}_{\text{450}}\text{ of the treated group}\\ -\;{\text{OD}}_{\text{450}}\text{ of the blank wells})\text{/}({\text{OD}}_{\text{450}}\text{ of the control group}\\ -\;{\text{OD}}_{\text{450}}\text{ of the blank wells})]\;\times \text{ 100}\% \end{array}$$

Each group included five technical replicates in each experiment, and three independent experiments were performed. Technical replicates were averaged within each experiment, and the mean value from each independent experiment was used as one statistical replicate.

### 2.18 RNA extraction and RT-qPCR

Total RNA was extracted from the treated cells using TRIzol reagent, and RNA concentration and purity were measured. Equal amounts of RNA were reverse-transcribed into complementary DNA (cDNA), followed by quantitative polymerase chain reaction. β-Actin was used as the internal reference gene, and relative FN1 mRNA expression was calculated using the 2^−ΔΔ*Ct*^ method.

RT-qPCR was used to evaluate the effects of different BPA concentrations on FN1 expression and to compare the knockdown efficiencies of the three candidate siFN1 sequences.

### 2.19 Western blotting

After treatment, cells were washed with ice-cold phosphate-buffered saline (PBS) and lysed in radioimmunoprecipitation assay (RIPA) buffer. Total protein concentrations were determined using the bicinchoninic acid (BCA) assay. Equal amounts of protein were separated by sodium dodecyl sulfate–polyacrylamide gel electrophoresis (SDS-PAGE) and transferred onto polyvinylidene fluoride (PVDF) membranes.

After blocking, the membranes were incubated with primary antibodies against FN1, phosphoinositide 3-kinase (PI3K), phosphorylated PI3K (p-PI3K), protein kinase B (AKT), phosphorylated AKT (p-AKT), endothelial nitric oxide synthase (eNOS), phosphorylated eNOS (p-eNOS), cluster of differentiation 31 (CD31), vascular endothelial cadherin (VE-cadherin), neural cadherin (N-cadherin), vimentin, and β-actin.

The primary antibodies included FN1 (Proteintech, 66042-1-Ig), PI3K (Proteintech, 20584-1-AP), p-PI3K (Bioss, BS-6417R), AKT (Proteintech, 10176-2-AP), p-AKT (Proteintech, 66444-1-Ig), eNOS/NOS3 (Proteintech, 85020-4-RR), p-eNOS (Thermo Fisher Scientific, MA5-14957), CD31/PECAM1 (Proteintech, 85898-4-RR), VE-cadherin/CD144 (Proteintech, 32332-1-AP), N-cadherin (Proteintech, 22018-1-AP), vimentin (Proteintech, 60330-1-Ig), and β-actin (Proteintech, 66009-1-Ig). Primary antibodies were diluted according to the manufacturers' recommendations. The membranes were then incubated with the corresponding horseradish peroxidase (HRP)-conjugated secondary antibodies, and protein bands were visualized using enhanced chemiluminescence.

Western blot band intensities were quantified using ImageJ software (version 1.49; National Institutes of Health, Bethesda, MD, USA). FN1, CD31, VE-cadherin, N-cadherin, and vimentin levels were normalized to β-actin. The phosphorylation status of the PI3K/AKT/eNOS pathway was expressed as the p-PI3K/PI3K, p-AKT/AKT, and p-eNOS/eNOS ratios.

### 2.20 Statistical analysis

Statistical analyses and data visualization were performed using R software (version 4.4.2; R Foundation for Statistical Computing, Vienna, Austria). Differentially expressed genes in the self-generated transcriptomic dataset were identified using the prespecified criteria of |log_2_ fold change| >1 and nominal *P* < 0.05, as described in Section 2.3. Multiple testing in the GO and KEGG enrichment analyses was corrected using the Benjamini–Hochberg method, and an adjusted *P* value < 0.05 was considered statistically significant.

The EndMT-like remodeling scores of FN1-high and FN1-low endothelial cells were compared using the Wilcoxon rank-sum test. The spot-level association between FN1 expression and the EndMT-like remodeling score was assessed using Spearman correlation analysis, and the scores of FN1-high and FN1-low spatial spots were compared using the Wilcoxon rank-sum test.

All *in vitro* experiments were independently repeated three times using separately cultured and treated cells, and each independent experiment was considered one biological replicate. For the CCK-8 assay, each condition included five technical wells within each independent experiment. Technical replicates were averaged within each experiment and were not treated as independent statistical observations. *In vitro* data were analyzed using GraphPad Prism (version 10.1.2; GraphPad Software, LLC, Boston, MA, USA) and are presented as the mean ± standard deviation of the three independent experiments. RT-qPCR data from the BPA concentration experiment were analyzed using one-way analysis of variance (ANOVA), followed by Dunnett's multiple-comparison test, with each BPA concentration compared with the 0 μM group. Other multigroup *in vitro* experiments were analyzed using one-way ANOVA followed by Tukey's multiple-comparison test. Dunnett's and Tukey's procedures were used to control for multiple comparisons within the corresponding families of group comparisons. All statistical tests were two-sided, and *P* < 0.05 was considered statistically significant.

## 3 Results

### 3.1 Toxicity characteristics of BPA and identification of BPA–DMED-associated candidate genes

**Figure 1:**
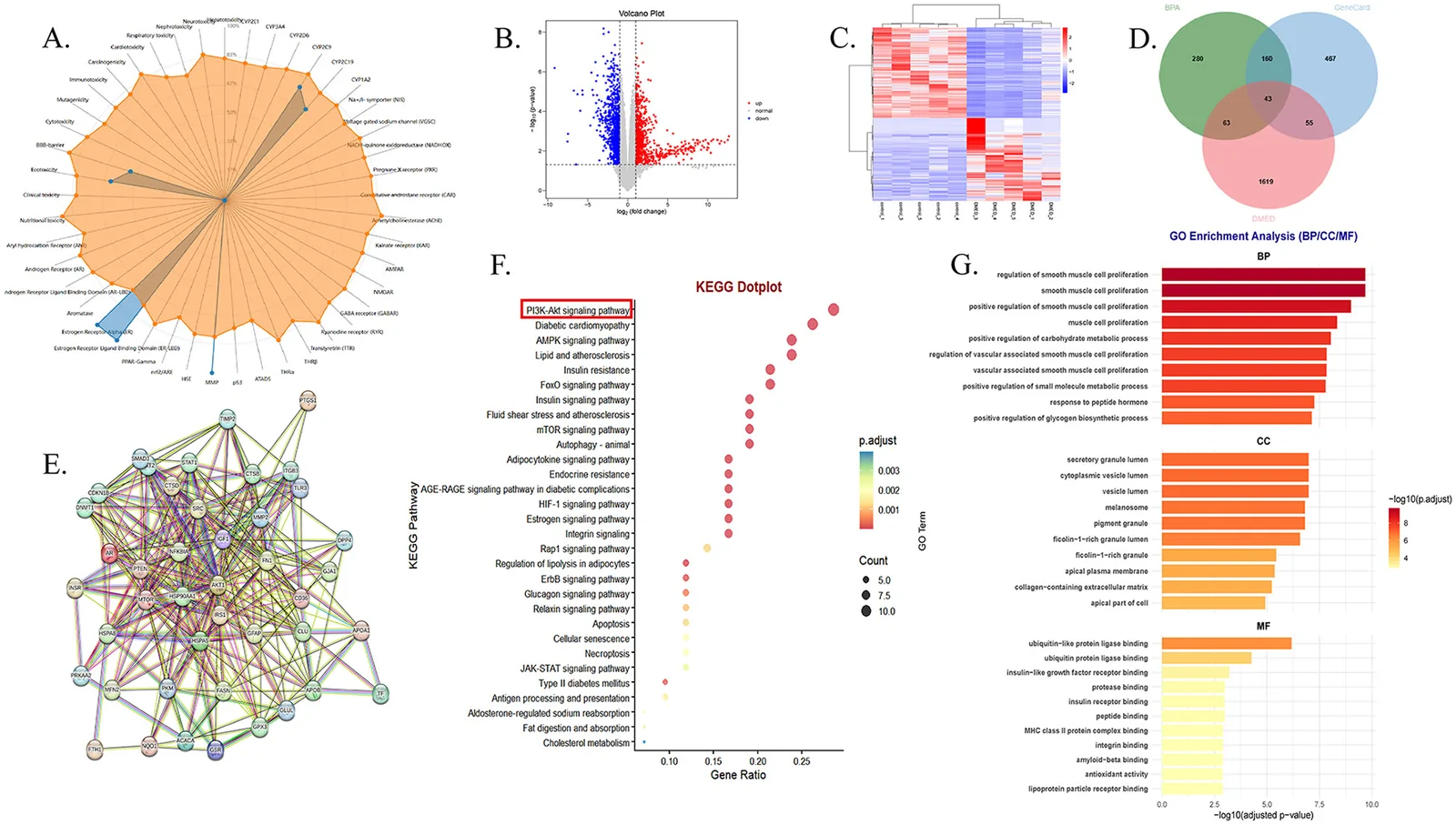
Toxicity profile of BPA and identification and functional characterization of BPA–DMED-associated candidate genes. **(A)** Predicted toxicity profile of BPA generated using ProTox-3.0. **(B)** Volcano plot of differentially expressed genes between the control and DMED groups in the self-generated rat corpus cavernosum transcriptomic dataset. Red and blue dots represent upregulated and downregulated genes, respectively, whereas gray dots represent genes without significant differential expression. **(C)** Heatmap of differentially expressed genes in the control and DMED samples. **(D)** Venn diagram showing the intersection among BPA targets, DMED-associated genes, and differentially expressed genes from the self-generated DMED dataset. A total of 43 shared candidate genes were identified. **(E)** PPI network of the 43 BPA–DMED-associated candidate genes. **(F)** KEGG enrichment analysis of the candidate genes. Dot size represents the number of enriched genes, and dot color represents the adjusted *P* value. **(G)** GO enrichment analysis of the candidate genes in the biological process, cellular component, and molecular function categories.

The chemical structure and basic physicochemical information of BPA were obtained from PubChem, and its potential toxicity profile was predicted using ProTox-3.0. BPA showed relatively high predicted toxicity for ecotoxicological endpoints. It was also predicted to interact with the estrogen receptor (ER) and its ligand-binding domain (ER-LBD) and to potentially affect mitochondrial membrane potential (MMP; Figure 1A and Table 1).

**Table 1:** Basic chemical and structural information of bisphenol A.

Name	SMILES structures	CAS	Molecular formula	Molecular weight
Bisphenol A	CC(C)(C1=CC=C(C=C1)O) C2=CC=C(C=C2)O	80-05-7	C_15_H_16_O_2_	228.29 g/mol

Differential expression analysis was subsequently performed using the self-generated transcriptomic data from the corpus cavernosum of DMED rats. The volcano plot showed multiple upregulated and downregulated genes in the DMED group compared with the control group (Figure 1B). The heatmap of DEGs showed distinct overall expression patterns between the control and DMED samples (Figure 1C).

Potential BPA targets were integrated from CTD, SwissTargetPrediction, and SEA, whereas DMED-associated genes were obtained from GeneCards. Intersection of the BPA targets, DMED-associated genes, and DEGs from the self-generated DMED dataset yielded 43 BPA–DMED-associated candidate genes (Figure 1D).

### 3.2 BPA–DMED-associated candidate genes are primarily involved in PI3K–Akt signaling and extracellular matrix-related processes

The 43 candidate genes were submitted to STRING to construct a PPI network. Most candidate proteins formed an interconnected network that included AKT1, MTOR, SRC, PTEN, FN1, and IGF1 (Figure 1E).

KEGG enrichment analysis showed that the candidate genes were mainly associated with the PI3K–Akt signaling pathway, AMPK signaling pathway, insulin resistance, FoxO signaling pathway, insulin signaling pathway, mTOR signaling pathway, AGE–RAGE signaling pathway in diabetic complications, HIF-1 signaling pathway, estrogen signaling pathway, and integrin-related signaling (Figure 1F). The PI3K–Akt signaling pathway showed the most prominent enrichment.

GO enrichment analysis showed that the candidate genes were involved in smooth muscle cell proliferation, vascular-associated smooth muscle cell proliferation, regulation of carbohydrate metabolism, and responses to hormones within the biological process category. Enriched cellular component terms included the secretory granule lumen, vesicle lumen, apical plasma membrane, and collagen-containing extracellular matrix. Enriched molecular function terms included integrin binding, insulin and insulin-like growth factor receptor binding, protease binding, and antioxidant activity (Figure 1G).

### 3.3 Integration of network topology and machine-learning-assisted feature selection prioritizes FN1

**Figure 2:**
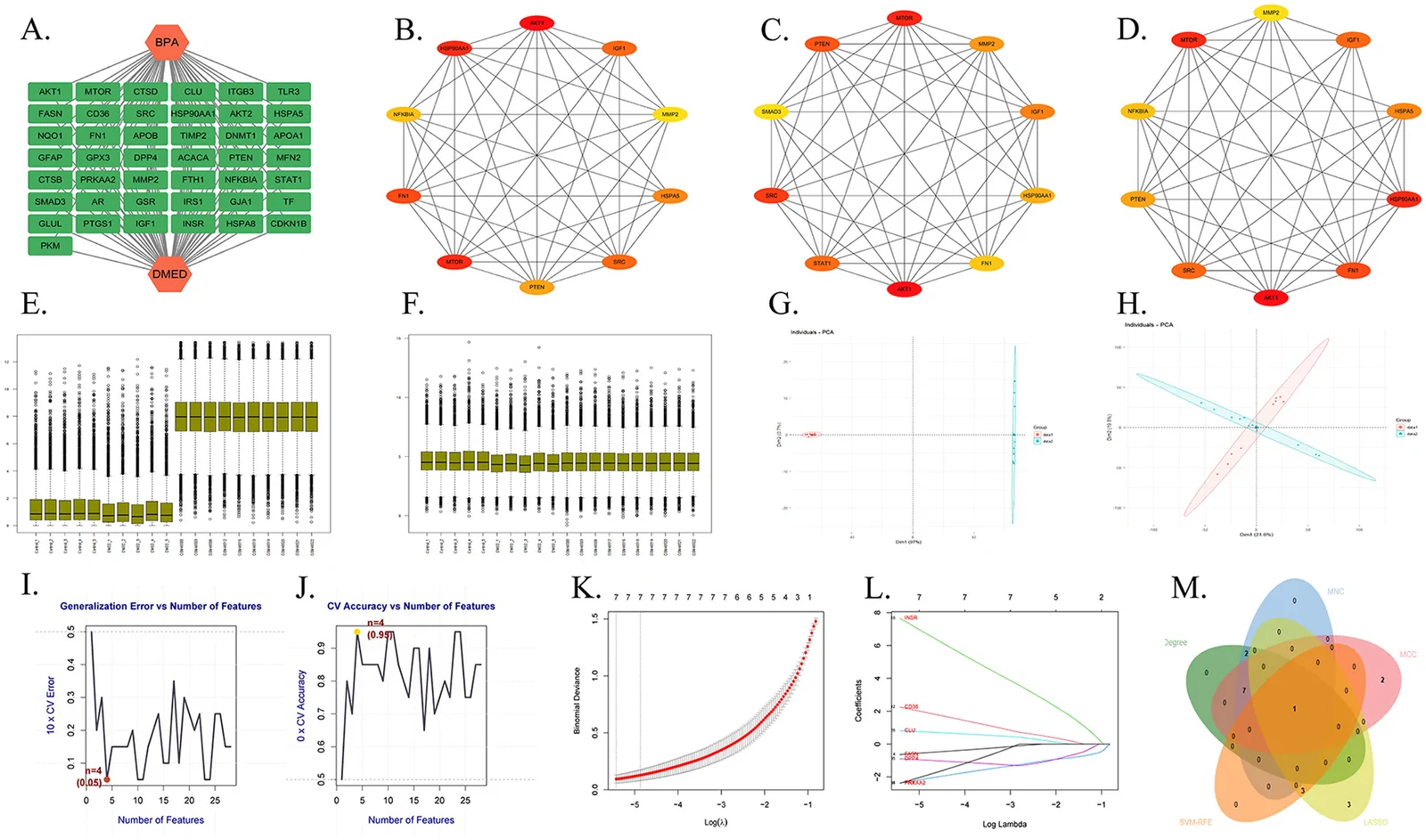
Integration of network topology and machine-learning-assisted feature selection prioritizes FN1. **(A)** BPA–candidate gene–DMED association network. **(B–D)** Top 10 hub genes identified using the MCC, MNC, and Degree algorithms, respectively. Node color represents the relative topological ranking. **(E, F)** Expression-distribution boxplots before and after ComBat batch correction, respectively. **(G, H)** PCA plots before and after batch correction, respectively. **(I)** Ten-fold cross-validation error across different feature-set sizes in the SVM-RFE analysis. **(J)** Ten-fold cross-validation accuracy across different feature-set sizes in the SVM-RFE analysis. **(K)** Cross-validation curve used to determine the optimal penalty parameter in the LASSO model. **(L)** LASSO coefficient profiles of the candidate genes. **(M)** Intersection of the genes identified by MCC, MNC, Degree, SVM-RFE, and LASSO, with FN1 identified as the only shared candidate gene.

A BPA–candidate gene–DMED association network was constructed, and the MCC, MNC, and Degree algorithms were used to identify the top 10 hub genes from each topological analysis (Figures 2A–D).

The self-generated DMED transcriptomic dataset was subsequently integrated with GSE2457. Before batch correction, samples from the two data sources showed marked differences in expression distributions and PCA space. After ComBat correction, the overall expression distributions became more comparable, and the separation attributable to dataset origin was reduced, while the distinction between the control and disease groups was retained (Figures 2E–H).

SVM-RFE and LASSO regression were then applied to the genes shared among the BPA targets, DMED-associated genes, and DEGs from the self-generated DMED dataset. SVM-RFE identified the optimal feature set according to cross-validation error, whereas LASSO retained genes with non-zero coefficients at the selected penalty parameter (Figures 2I–L). Integration of the MCC, MNC, Degree, SVM-RFE, and LASSO results identified FN1 as the only gene shared by all five approaches (Figure 2M). FN1 was therefore selected for subsequent single-cell, spatial transcriptomic, molecular simulation, and *in vitro* analyses.

### 3.4 Single-cell transcriptomics reveals heterogeneous FN1 expression across corpus cavernosum cell types

**Figure 3:**
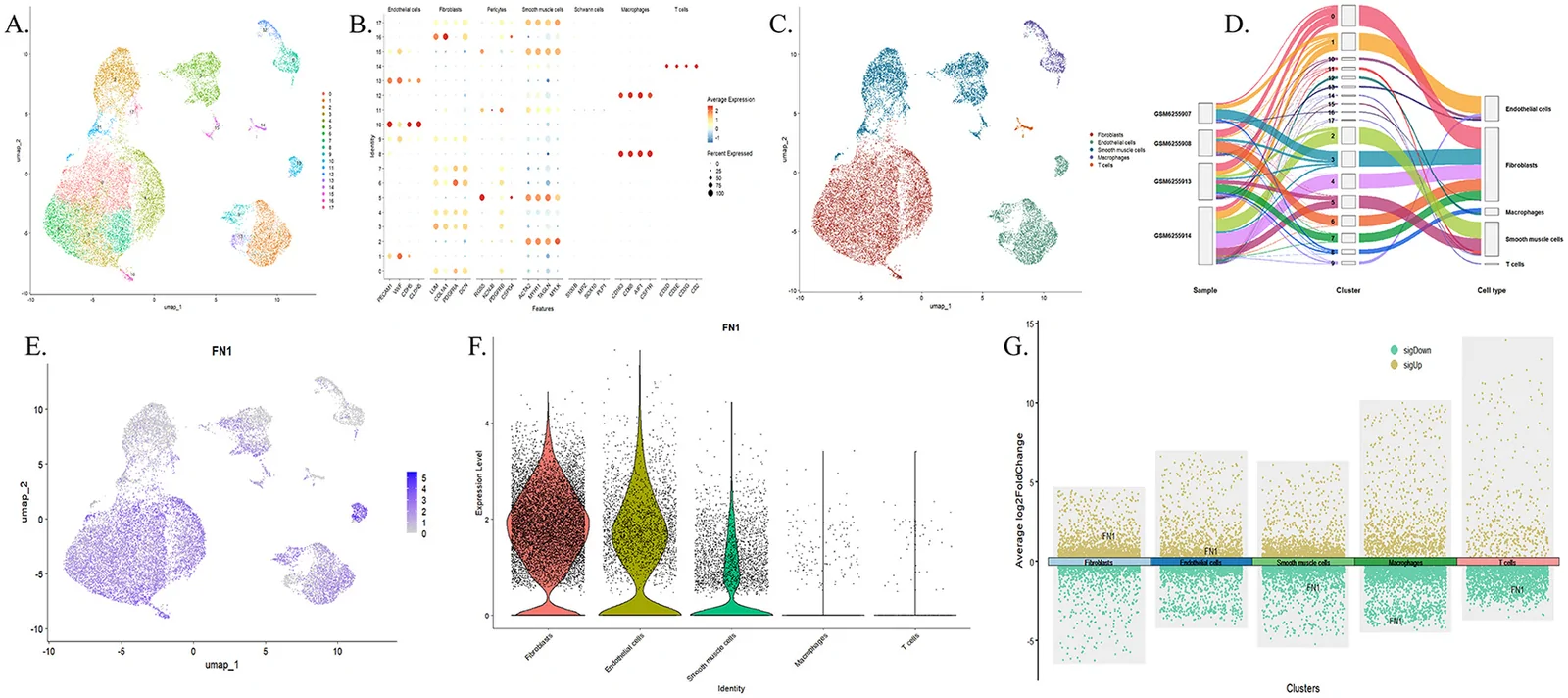
Single-cell transcriptomic profiling and cell-type-specific distribution of FN1 in the human corpus cavernosum. **(A)** UMAP visualization of the cell clusters identified after quality control, batch correction, and dimensionality reduction. **(B)** Dot plot showing the expression of canonical marker genes used to evaluate the candidate cell lineages. Dot size represents the percentage of cells expressing each marker, and dot color represents the average expression level. **(C)** UMAP visualization of the final annotated cell populations, including endothelial cells, fibroblasts, smooth muscle cells, macrophages, and T cells. **(D)** Alluvial plot showing the relationships among samples, cell clusters, and the final cell-type annotations. **(E)** Feature plot showing the distribution of FN1 expression across the annotated cell populations. **(F)** Violin plots showing FN1 expression across the major cell types. **(G)** Cell-type-specific differential-expression plots comparing DMED with normal samples, with FN1 highlighted within each cell type.

After quality control, batch correction, dimensionality reduction, and clustering of the GSE206528 single-cell RNA-sequencing dataset, multiple cell clusters were obtained (Figure 3A). Based on canonical marker-gene expression, the clusters were annotated as endothelial cells, fibroblasts, smooth muscle cells, macrophages, and T cells (Figures 3B–D).

FN1 expression was heterogeneous across cell types. UMAP visualization showed that FN1 was predominantly expressed in fibroblast-enriched regions and in a subset of endothelial cells (Figure 3E). FN1 expression was higher in fibroblasts and endothelial cells than in smooth muscle cells, macrophages, and T cells (Figure 3F). Cell-type-specific comparisons between the DMED and corresponding control groups showed higher FN1 expression in fibroblasts and endothelial cells, whereas lower expression was observed in smooth muscle cells, macrophages, and T cells (Figure 3G).

### 3.5 Pseudotemporal analysis shows distinct transcriptional-state distributions among DMED cell types

**Figure 4:**
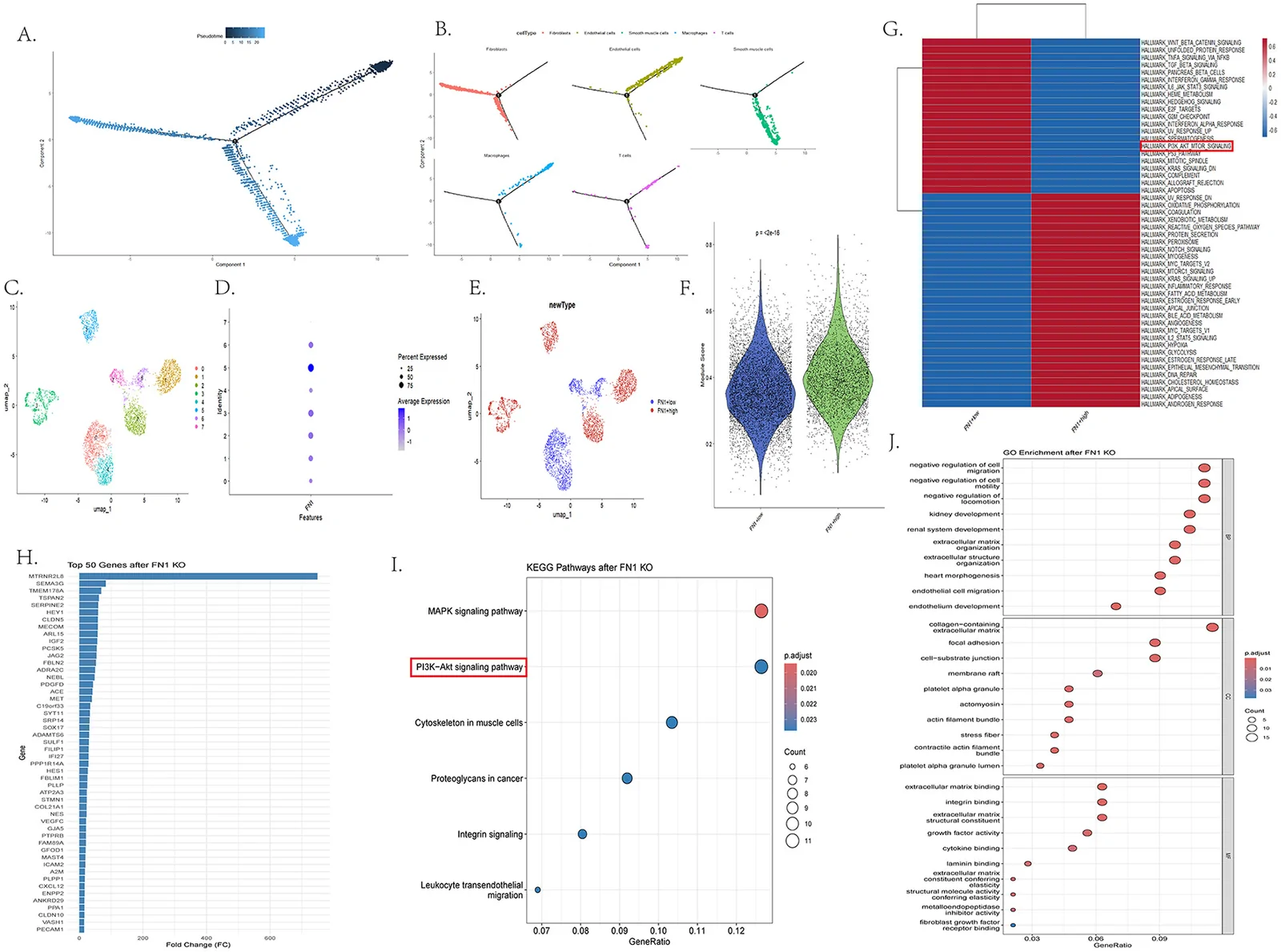
Pseudotemporal distribution, FN1-associated endothelial remodeling, and virtual FN1 perturbation analysis. **(A)** Pseudotemporal trajectory of all annotated cells from the DMED samples, colored according to inferred pseudotime. **(B)** Distribution of the major cell types along the inferred pseudotemporal trajectory. **(C)** UMAP visualization of eight endothelial cell subclusters identified after reclustering DMED endothelial cells. **(D)** Dot plot showing FN1 expression and the proportion of FN1-positive cells across the endothelial subclusters. **(E)** UMAP visualization of FN1-high and FN1-low DMED endothelial cells. **(F)** Comparison of the EndMT-like remodeling score between FN1-high and FN1-low endothelial cells. **(G)** GSVA heatmap showing differences in Hallmark pathway activity between FN1-high and FN1-low endothelial cells. **(H)** Top 50 predicted response genes following virtual FN1 perturbation, ranked according to the estimated perturbation effect. **(I)** KEGG enrichment analysis of the genes predicted to respond to virtual FN1 perturbation. **(J)** GO enrichment analysis of the predicted response genes in the biological process, cellular component, and molecular function categories. The virtual perturbation results represent computational predictions and should not be interpreted as evidence from an actual FN1 knockout experiment.

Pseudotemporal analysis was performed using all annotated cells from the DMED samples. Cells were distributed along multiple branches and assigned to different inferred pseudotemporal states (Figure 4A). The major cell types occupied distinct regions of the trajectory. Endothelial cells were predominantly located near the inferred starting region, whereas fibroblasts were enriched toward the terminal regions of two branches. Smooth muscle cells, macrophages, and T cells were distributed across other regions of the trajectory (Figure 4B).

These results describe differences in the inferred transcriptional-state distributions of the major cell types in DMED tissue. The trajectory represents a computational ordering based on transcriptomic similarity and does not establish a true lineage relationship or direct conversion of endothelial cells into fibroblasts.

### 3.6 FN1-high endothelial cells exhibit enhanced EndMT-like remodeling features

Endothelial cells from the DMED samples were further extracted and reclustered, yielding eight endothelial subclusters (Figure 4C). Both FN1 expression levels and the proportions of FN1-positive cells varied among the endothelial subclusters (Figure 4D).

DMED endothelial cells were divided into FN1-high and FN1-low groups according to the median FN1 expression. The two groups showed different distributions in UMAP space (Figure 4E). The EndMT-like remodeling score was significantly higher in FN1-high endothelial cells than in FN1-low endothelial cells (Figure 4F).

GSVA revealed distinct pathway-activity patterns between FN1-high and FN1-low endothelial cells. Compared with FN1-low cells, FN1-high cells showed lower relative enrichment of PI3K/AKT/mTOR signaling. In contrast, oxidative stress-, hypoxia-, glycolysis-, angiogenesis-, and mesenchymal transition-related programs showed higher relative scores in FN1-high cells (Figure 4G). These findings indicate that the FN1-high endothelial state was characterized by enhanced stress and remodeling features accompanied by reduced PI3K/AKT/mTOR pathway activity.

### 3.7 Virtual FN1 perturbation predicts involvement in PI3K–Akt, integrin, and extracellular matrix-related networks

Virtual FN1 perturbation was performed in DMED endothelial cells using scTenifoldKnk. Genes predicted to respond to FN1 perturbation were mainly associated with cellular stress responses, cell adhesion, cell migration, and signal transduction (Figure 4H).

KEGG enrichment analysis showed that the predicted response genes were associated with the MAPK signaling pathway, PI3K–Akt signaling pathway, cytoskeleton-related pathways, proteoglycans in cancer, integrin-related signaling, and leukocyte transendothelial migration (Figure 4I).

GO enrichment analysis showed that these genes were involved in the regulation of cell migration and motility, extracellular matrix organization, endothelial cell migration and development, cell–matrix junctions, focal adhesions, and extracellular matrix structural constituent binding (Figure 4J). These results represent computational predictions of transcriptional responses to virtual FN1 perturbation.

### 3.8 Normal human spatial reference analysis shows a spatial association between FN1 and EndMT-like remodeling

**Figure 5:**
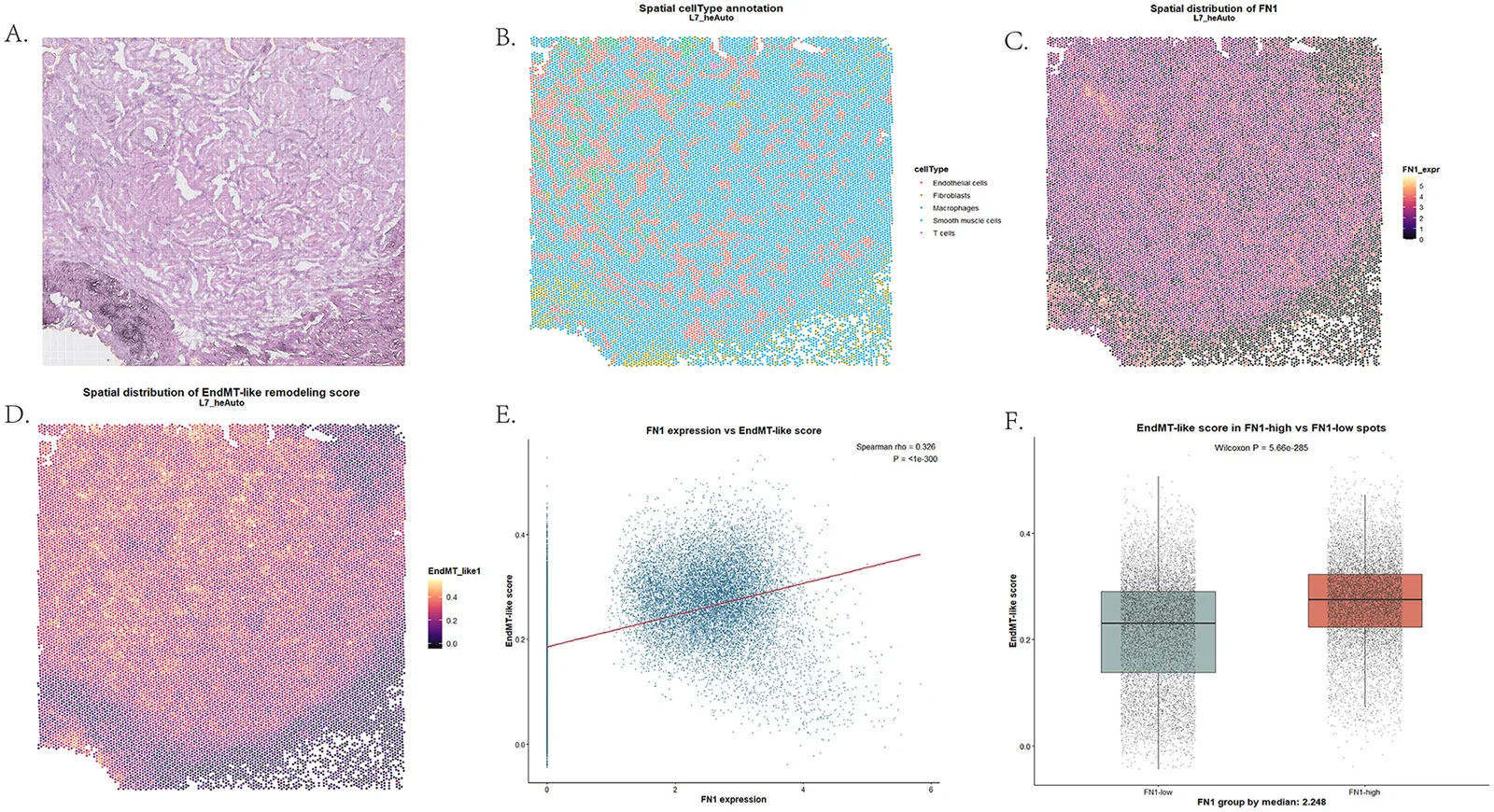
Spatial reference analysis of FN1-associated EndMT-like remodeling in the normal human corpus cavernosum. **(A)** Histological image of the L7_heAuto region from the normal human corpus cavernosum spatial transcriptomic dataset. **(B)** Spatial distribution of the predicted predominant cell-type signatures inferred by label transfer from the single-cell reference dataset. **(C)** Spatial distribution of FN1 expression. **(D)** Spatial distribution of the EndMT-like remodeling score. **(E)** Spot-level correlation between FN1 expression and the EndMT-like remodeling score. The red line represents the fitted trend; Spearman ρ = 0.326, *P* < 0.001. **(F)** Comparison of the EndMT-like remodeling score between FN1-high and FN1-low spots classified according to median FN1 expression. All spatial spots were derived from a single normal human sample; therefore, the analysis provides a descriptive anatomical reference and does not indicate DMED-specific spatial alterations.

Spatial transcriptomic data from the normal human corpus cavernosum dataset GSE261085 were analyzed to examine the spatial organization of FN1-associated remodeling features. Histological imaging showed that the selected region contained cavernous trabeculae, vascular spaces, and stromal structures (Figure 5A).

Following label transfer from the single-cell reference dataset, spatial spots predominantly exhibited smooth muscle cell-, endothelial cell-, and fibroblast-related signatures, together with smaller regions displaying macrophage- and T-cell-related signals (Figure 5B). FN1 expression showed spatial heterogeneity and was relatively enriched in selected stromal and trabecular regions (Figure 5C). The EndMT-like remodeling score also showed a regional distribution (Figure 5D).

At the spot level, FN1 expression was positively correlated with the EndMT-like remodeling score (Spearman ρ = 0.326, *P* < 0.001; Figure 5E). FN1-high spots showed significantly higher EndMT-like remodeling scores than FN1-low spots (Wilcoxon *P* < 0.001; Figure 5F).

These findings show a descriptive spot-level association between FN1 expression and EndMT-like remodeling activity in the normal human corpus cavernosum reference dataset. Because all spatial spots were derived from a single normal human sample, these results do not indicate DMED-specific spatial alterations.

### 3.9 Molecular docking and molecular dynamics simulations suggest potential structural compatibility between BPA and an FN1 domain

**Figure 6:**
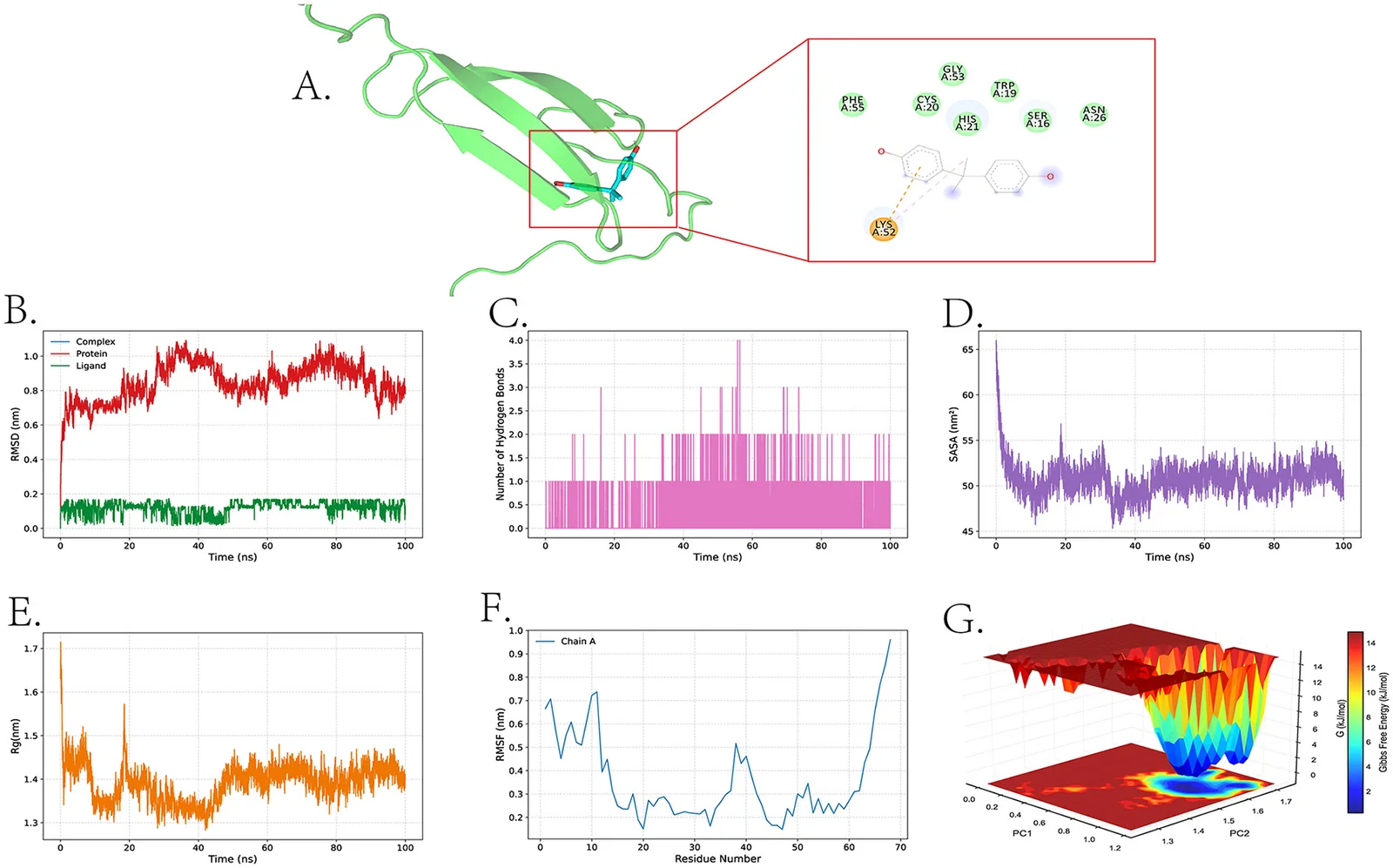
Molecular docking and molecular dynamics analysis of the computationally modeled BPA–FN1 system. **(A)** Predicted three-dimensional docking pose and corresponding two-dimensional contact map of BPA within the available FN1 domain. **(B)** RMSD trajectories of the BPA–FN1 complex, FN1 protein, and BPA ligand during the 100-ns molecular dynamics simulation. **(C)** Number of protein–ligand hydrogen bonds throughout the simulation. **(D)** SASA of the FN1 domain during the simulation. **(E)** Rg of the FN1 domain during the simulation. **(F)** Residue-level RMSF of FN1. **(G)** Free-energy landscape of the BPA–FN1 complex based on the first two principal components.

Molecular docking predicted a potential pose in which BPA may occupy a region within the available FN1 domain and form non-covalent contacts with surrounding amino acid residues, including hydrophobic and van der Waals interactions (Figure 6A).

During the 100-ns MD simulation, the BPA–FN1 complex underwent an initial conformational adjustment, followed by reduced fluctuation during the later phase of the trajectory. The RMSD of the complex remained within a relatively narrow range during the latter part of the simulation (Figure 6B). The number of protein–ligand hydrogen bonds fluctuated throughout the simulation (Figure 6C). SASA and Rg remained within limited ranges, indicating no marked changes in the overall solvent exposure or compactness of the FN1 domain (Figures 6D, E). RMSF analysis showed that greater fluctuations were mainly restricted to local flexible regions, whereas most residues showed relatively limited fluctuations (Figure 6F). The free-energy landscape contained a concentrated low-energy region sampled during the simulation (Figure 6G).

MM/GBSA analysis yielded an estimated binding free energy of −25.24 kcal/mol for the BPA–FN1 complex (Table 2). Van der Waals, electrostatic, and non-polar solvation components contributed favorably to the estimated binding energy, whereas polar solvation opposed complex formation.

**Table 2:** MM/GBSA binding free-energy components of the BPA–FN1 complex.

Component	Energy (kcal/mol)	Std. Dev.	Meaning
Delta E_vdw	−23.18	3.53	Van der Waals interaction
Delta E_ele	−4.33	0.64	Electrostatic interaction
Delta G_polar	4.99	0.73	Polar solvation energy
Delta G_nonpolar	−2.72	0.32	Non-polar solvation energy
Delta G_bind (Total)	−25.24	3.36	Total binding free energy

### 3.10 Structure-based screening prioritizes baicalin as a candidate FN1-associated intervention compound

**Figure 7:**
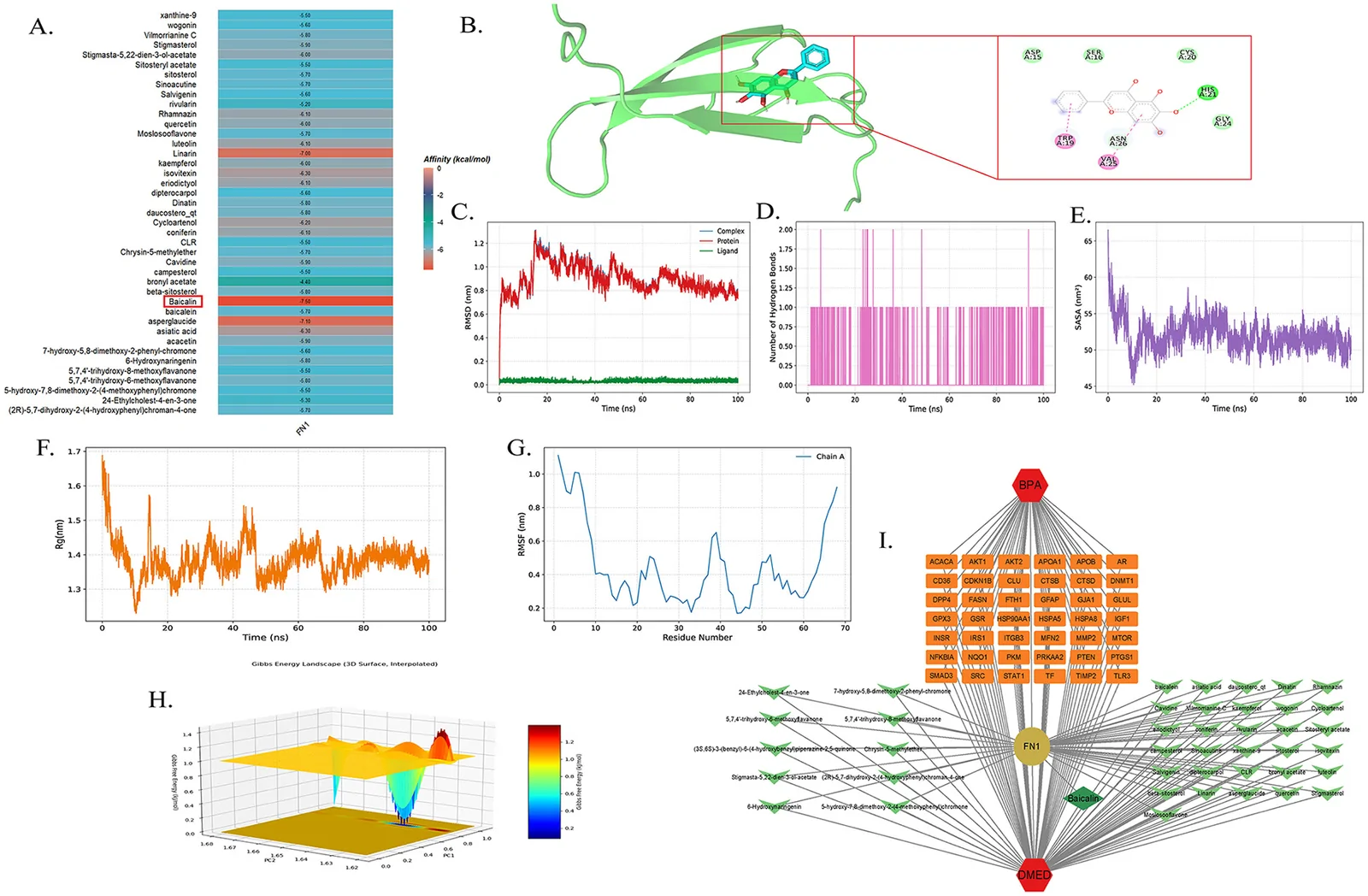
Structure-based screening and molecular simulation of baicalin as a candidate FN1-associated compound. **(A)** Predicted binding energies of the FN1-associated plant-derived candidate compounds obtained by batch blind docking. Baicalin is highlighted. **(B)** Predicted three-dimensional docking pose and corresponding two-dimensional contact map of baicalin within the available FN1 domain. **(C)** RMSD trajectories of the baicalin–FN1 complex, FN1 protein, and baicalin ligand during the 100-ns molecular dynamics simulation. **(D)** Number of protein–ligand hydrogen bonds throughout the simulation. **(E)** SASA of the FN1 domain during the simulation. **(F)** Rg of the FN1 domain during the simulation. **(G)** Residue-level RMSF of FN1. **(H)** Free-energy landscape of the baicalin–FN1 complex based on the first two principal components. **(I)** BPA–candidate gene–DMED–FN1–phytochemical association network illustrating the relationship among BPA, the candidate genes, FN1, DMED, and the screened plant-derived compounds.

FN1-associated plant-derived candidate compounds collected from HERB 2.0 were subjected to batch blind docking using AutoDock Vina. The screened compounds showed different predicted binding energies toward the FN1 domain. Baicalin had a predicted docking energy of −7.50 kcal/mol and ranked among the compounds with relatively favorable docking results (Figure 7A).

Based on the docking ranking, baicalin was selected for subsequent MD simulations and *in vitro* evaluation. The BPA–candidate gene–FN1–phytochemical network showed the relationship among baicalin, FN1, and the BPA–DMED candidate network (Figure 7I).

### 3.11 The predicted baicalin–FN1 docking pose exhibits relatively stable simulated dynamics

Molecular docking predicted a potential pose in which baicalin may occupy a region within the available FN1 domain and form non-covalent contacts with TRP19, HIS21, TYR25, ASN26, and surrounding residues, including predicted hydrogen bonds, hydrophobic contacts, and van der Waals contacts (Figure 7B).

During the 100-ns MD simulation, the baicalin–FN1 complex underwent an initial conformational adjustment, followed by reduced fluctuation during the later phase of the trajectory. The RMSD values of the protein and complex remained relatively stable during the latter part of the simulation, whereas the ligand showed limited fluctuation within the predicted binding region (Figure 7C). Protein–ligand hydrogen bonds were intermittently maintained throughout the trajectory, with approximately one hydrogen bond observed during much of the simulation and occasional increases to two hydrogen bonds (Figure 7D).

SASA decreased during the early phase of the simulation and subsequently remained within a relatively narrow range (Figure 7E). Rg stabilized after the initial adjustment, with no marked increase in overall protein dimensions (Figure 7F). RMSF analysis showed relatively limited fluctuations for most residues, with greater flexibility mainly observed at the termini and selected local regions (Figure 7G). The free-energy landscape contained a concentrated low-energy region sampled during the simulation (Figure 7H).

MM/GBSA analysis yielded an estimated binding free energy of −29.35 kcal/mol for the baicalin–FN1 complex (Table 3). This value was more negative than that estimated for the BPA–FN1 complex under the same computational conditions.

**Table 3:** MM/GBSA binding free energy components of the baicalin–FN1 complex.

Component	Energy (kcal/mol)	Std. Dev.	Meaning
Delta E_vdw	−27.97	1.83	Van der Waals interaction
Delta E_ele	−4.27	0.94	Electrostatic interaction
Delta G_polar	5.81	0.6	Polar solvation energy
Delta G_nonpolar	−2.92	0.13	Non-polar solvation energy
Delta G_bind (Total)	−29.35	1.79	Total binding free energy

### 3.12 BPA induces FN1 upregulation, EndMT-like phenotypic alterations, and PI3K/AKT/eNOS signaling dysregulation

**Figure 8:**
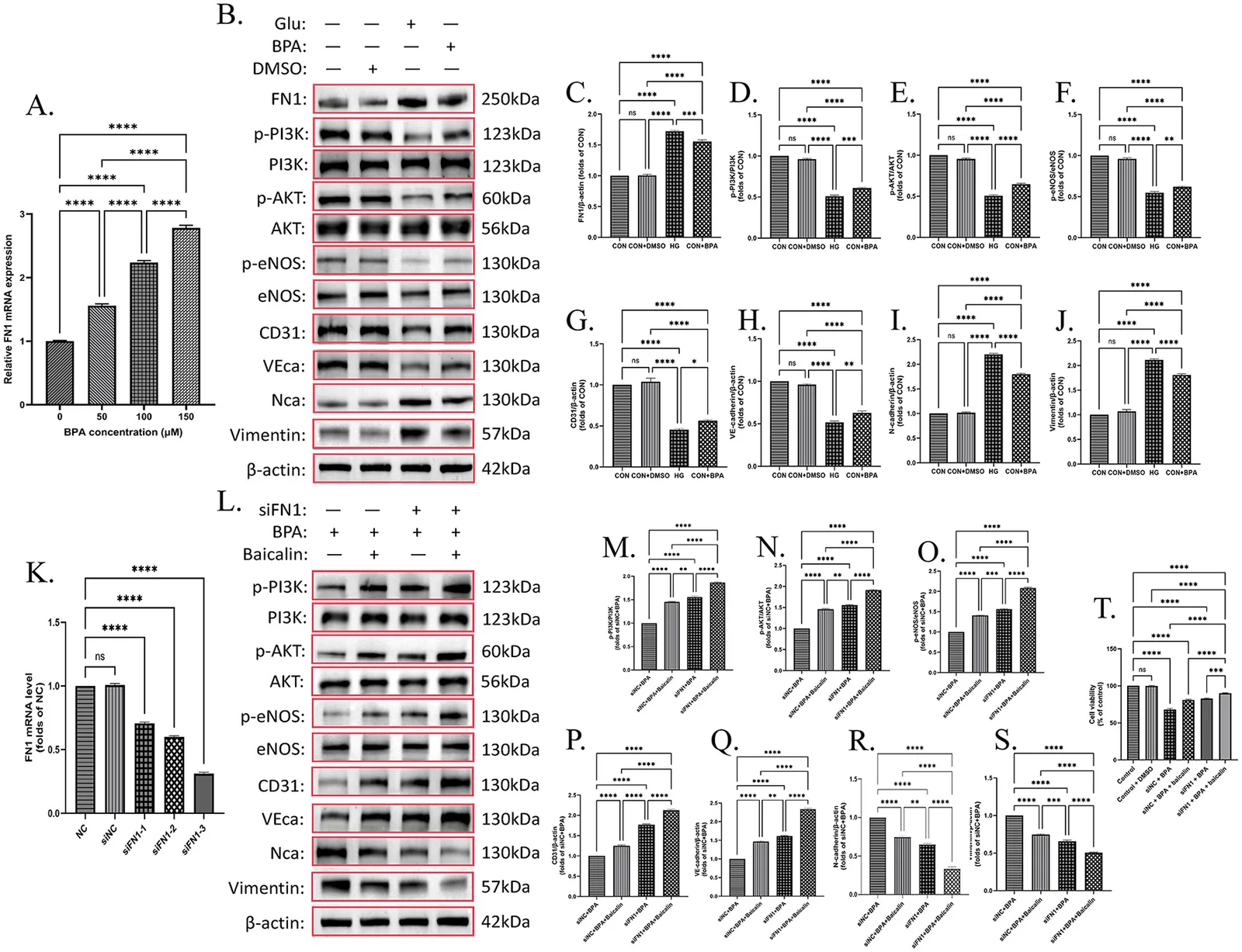
Effects of BPA exposure, FN1 knockdown, and delayed baicalin intervention on endothelial remodeling and PI3K/AKT/eNOS signaling. **(A)** Relative FN1 mRNA expression in corpus cavernosum endothelial cells treated with 0, 50, 100, or 150 μM BPA for 24 h. **(B)** Representative Western blot images of FN1, PI3K/AKT/eNOS pathway proteins, endothelial markers, and mesenchymal-related markers in the control, control+DMSO, HG, and BPA groups. **(C–J)** Quantification of FN1/β-actin, p-PI3K/PI3K, p-AKT/AKT, p-eNOS/eNOS, CD31/β-actin, VE-cadherin/β-actin, N-cadherin/β-actin, and vimentin/β-actin, respectively. **(K)** Relative FN1 mRNA expression after transfection with siNC or three candidate siFN1 sequences. siFN1-3 showed the greatest knockdown efficiency and was selected for subsequent experiments. **(L)** Representative Western blot images from the siNC+BPA, siNC+BPA+baicalin, siFN1+BPA, and siFN1+BPA+baicalin groups. **(M–S)** Quantification of p-PI3K/PI3K, p-AKT/AKT, p-eNOS/eNOS, CD31/β-actin, VE-cadherin/β-actin, N-cadherin/β-actin, and vimentin/β-actin, respectively. **(T)** Cell viability measured using the CCK-8 assay in the control, control+DMSO, siNC+BPA, siNC+BPA+baicalin, siFN1+BPA, and siFN1+BPA+baicalin groups. Data are presented as the mean ± SD from three independent experiments. β-Actin was used as the loading control for FN1 and the endothelial and mesenchymal-related markers, whereas phosphorylated proteins were normalized to their corresponding total proteins. ns, not significant; *P < 0.05; **P < 0.01; ***P < 0.001; ****P < 0.0001.

RT-qPCR showed that FN1 mRNA expression increased with increasing BPA concentrations after 24 h of exposure. Compared with the 0 μM group, FN1 expression was increased in the 50, 100, and 150 μM BPA groups, with the greatest increase observed at 150 μM (Figure 8A).

The control, control+DMSO, HG, and BPA groups were subsequently used to evaluate BPA-induced endothelial alterations. No significant differences were observed between the control and control+DMSO groups. Compared with the control group, FN1 protein expression was significantly increased in both the HG and BPA groups (Figures 8B, C).

Total PI3K, AKT, and eNOS protein levels showed no marked changes, whereas the p-PI3K/PI3K, p-AKT/AKT, and p-eNOS/eNOS ratios were significantly reduced in the HG and BPA groups (Figures 8D–F).

CD31 and VE-cadherin expression was significantly decreased, whereas N-cadherin and vimentin expression was significantly increased in the HG and BPA groups (Figures 8G–J). The direction of the BPA-induced changes was generally consistent with that observed following HG stimulation.

### 3.13 FN1 knockdown and delayed baicalin intervention partially attenuate BPA-induced endothelial abnormalities

The knockdown efficiencies of three candidate siFN1 sequences were first compared. RT-qPCR showed that all three siFN1 sequences reduced FN1 mRNA expression to varying degrees. siFN1-3 produced the greatest reduction and was selected for subsequent experiments (Figure 8K).

Under continuous BPA exposure, delayed baicalin intervention and FN1 knockdown significantly increased the p-PI3K/PI3K, p-AKT/AKT, and p-eNOS/eNOS ratios compared with the siNC+BPA group, whereas total PI3K, AKT, and eNOS protein levels showed no significant differences among the groups (Figures 8L–O).

Delayed baicalin intervention and FN1 knockdown significantly increased CD31 and VE-cadherin expression and reduced N-cadherin and vimentin expression compared with the siNC+BPA group (Figures 8P–S). The siFN1+BPA+baicalin group showed the greatest overall improvement among the intervention groups. This pattern does not indicate that all effects of baicalin were dependent on FN1.

The CCK-8 assay showed no significant difference in cell viability between the control and control+DMSO groups. Cell viability was significantly reduced in the siNC+BPA group. Compared with the siNC+BPA group, cell viability was significantly increased in the siNC+BPA+baicalin and siFN1+BPA groups, with the highest value observed in the siFN1+BPA+baicalin group (Figure 8T).

These findings show that delayed baicalin addition after the initiation of BPA exposure and siRNA-mediated FN1 knockdown both partially attenuated BPA-associated reductions in cell viability, EndMT-like phenotypic alterations, and PI3K/AKT/eNOS signaling abnormalities.

## 4 Discussion

BPA is widely used in the production of polycarbonate plastics and epoxy resins, and food containers, packaging materials, and can linings represent important sources of exposure in the general population (1, 2). BPA is also present in thermal paper, dust, water, and other environmental media, resulting in long-term exposure through dietary intake, inhalation, and dermal contact. Although the use of BPA in certain consumer products has been increasingly restricted in several countries and regions, concerns remain because of its extensive historical use, continued environmental presence, and the uncertain safety of some BPA substitutes (27). Epidemiological and experimental studies have linked BPA exposure to insulin resistance, disturbances in glucose and lipid metabolism, vascular endothelial dysfunction, and adverse male reproductive effects (3, 4, 28). These effects overlap with several pathological features of DMED, including metabolic stress, impaired endothelial NO signaling, and structural remodeling of the corpus cavernosum. Therefore, investigating the effects of BPA on cavernous endothelial homeostasis may provide insight into how environmental exposure contributes to endothelial abnormalities relevant to erectile dysfunction.

By integrating network toxicology, transcriptomic data, network topology, and machine-learning-assisted feature selection, the present study prioritized FN1 as a candidate molecule at the intersection of BPA-associated targets and DMED-related molecular alterations. Subsequent analyses showed that FN1 was mainly expressed in fibroblasts and a subset of endothelial cells in the corpus cavernosum and was associated with increased EndMT-like remodeling activity. *In vitro*, BPA exposure was accompanied by FN1 upregulation, reduced endothelial marker expression, increased mesenchymal-related marker expression, and decreased activation of PI3K/AKT/eNOS signaling. FN1 knockdown partially attenuated these changes, whereas delayed baicalin intervention also produced protective effects after BPA exposure had already begun. Collectively, these findings suggest that FN1-associated extracellular matrix and endothelial phenotypic remodeling may represent one component of BPA-related cavernous endothelial injury. They do not, however, establish that BPA directly causes or aggravates DMED. The novelty of the present study should not be interpreted as the identification of FN1 as a previously unknown fibrosis-associated molecule. Fibronectin has already been implicated in extracellular matrix accumulation and structural remodeling in diabetic penile tissue. Rather, the contribution of this study lies in placing FN1 within a specific environmental-exposure and cell-state context. FN1 was not selected solely on the basis of its known biological functions; it was the only candidate jointly retained by three network-topology algorithms and two machine-learning approaches. Subsequent single-cell analysis localized FN1 to fibroblasts and a subset of DMED endothelial cells, in which high FN1 expression was associated with an enhanced remodeling program. Moreover, siRNA-mediated FN1 knockdown partially attenuated BPA-associated endothelial-marker loss, mesenchymal-marker induction, and PI3K/AKT/eNOS signaling abnormalities. These findings extend previous observations of FN1 as a fibrosis-related marker by supporting its potential contribution to BPA-associated cavernous endothelial remodeling. Nevertheless, the prioritization of FN1 does not imply that it is the only or central mediator of BPA toxicity. The partial rescue observed after FN1 knockdown supports a contributory role for FN1 but does not establish that FN1 is necessary or sufficient for the full spectrum of BPA-induced endothelial abnormalities. Moreover, the upstream mechanism through which BPA increases FN1 expression was not resolved in the present study. BPA may influence FN1 expression or matrix assembly through endocrine-receptor signaling, oxidative stress, inflammatory responses, TGF-β-related pathways, or other stress-responsive mechanisms, but these possibilities were not experimentally distinguished. Therefore, the present findings identify FN1 as a data-prioritized candidate contributor to BPA-associated endothelial remodeling rather than establishing a unique BPA–FN1 regulatory pathway.

Erectile dysfunction is a multifactorial clinical disorder involving the coordinated regulation of vascular, neurological, endocrine, metabolic, and psychogenic processes. Clinical observations in patients with obstructive sleep apnea further illustrate this complexity, as the occurrence of erectile dysfunction was associated with disease severity, age, body mass index, oxygenation indices, and anxiety or depression (29). Accordingly, FN1-associated endothelial remodeling should be interpreted as a potential vascular component of BPA-related erectile impairment rather than as a sufficient explanation for erectile dysfunction as a whole. BPA may also influence erectile-function-related processes through endocrine disruption, oxidative and inflammatory stress, metabolic dysregulation, neural abnormalities, or alterations in cavernous smooth muscle homeostasis. However, these mechanisms and their relative contributions were not directly evaluated in the present study. Therefore, the current findings provide mechanistic plausibility for one endothelial pathway but do not establish clinical causality or quantify its contribution to the overall erectile dysfunction phenotype.

FN1 is a major extracellular matrix glycoprotein composed of multiple functional domains capable of interacting with integrins, collagen, and other matrix components (30, 31). It plays important roles in cell adhesion, migration, tissue repair, and matrix assembly. Under physiological conditions, FN1-containing matrix networks help maintain tissue architecture and cell–matrix communication. In diabetes, chronic inflammation, and persistent tissue injury, however, excessive FN1 expression and deposition are commonly associated with extracellular matrix expansion, altered matrix stiffness, and progressive fibrosis (31, 32). High-glucose conditions can promote FN1 production in vascular and stromal cells and alter integrin- and focal adhesion-related signaling, thereby shifting a transient reparative matrix response toward persistent pathological remodeling (33). In the corpus cavernosum, excessive matrix accumulation may modify the mechanical microenvironment surrounding endothelial and smooth muscle cells, disrupt sinusoidal architecture, and impair cell–cell and cell–matrix communication. Such changes may be unfavorable for sinusoidal expansion, smooth muscle relaxation, and blood filling during erection.

The association between FN1 and endothelial phenotypic remodeling is also biologically plausible. EndMT is a dynamic process involving coordinated changes in endothelial junctions, cellular polarity, migratory behavior, and extracellular matrix production rather than a simple replacement of molecular markers (34). During this process, endothelial cells progressively lose endothelial features such as CD31 and VE-cadherin while acquiring vimentin, N-cadherin, and extracellular matrix-related molecules. FN1 may serve both as a component of this remodeling state and as a regulator of cell behavior through integrin-, focal adhesion-, and cytoskeleton-related signaling (13, 34). In the present study, the high expression of FN1 in fibroblasts was consistent with their established role as matrix-producing cells, whereas its increase in a subset of endothelial cells suggested that endothelial cells themselves may also participate in abnormal matrix-related programs. FN1-high endothelial cells exhibited higher EndMT-like remodeling scores but lower relative PI3K/AKT/mTOR pathway activity. This pattern suggests that high FN1 expression marks an endothelial state characterized by enhanced matrix remodeling and phenotypic instability rather than activation of endothelial-protective signaling. In addition, FN1 expression was positively associated with the EndMT-like remodeling score in the normal human spatial reference dataset. These findings support an association between FN1 expression and endothelial phenotypic remodeling but do not establish a lineage transition. Because lineage tracing was not performed and the available evidence was based on transcriptional features, the term “EndMT-like remodeling” is more appropriate than complete and irreversible EndMT.

PI3K/AKT/eNOS signaling is an important molecular axis linking endothelial survival, vasodilation, and erectile function. AKT-mediated phosphorylation of eNOS promotes NO production, which activates soluble guanylate cyclase in cavernous smooth muscle cells, increases cGMP levels, and induces smooth muscle relaxation and cavernous engorgement (11). Oxidative stress, advanced glycation end products, and chronic inflammation in diabetes can reduce AKT/eNOS activation and NO bioavailability, contributing to endothelial dysfunction in DMED (10, 35). In the single-cell analysis, FN1-high endothelial cells showed lower relative PI3K/AKT/mTOR pathway activity, whereas the *in vitro* experiments showed that BPA-induced FN1 upregulation was accompanied by reduced phosphorylation of PI3K, AKT, and eNOS. FN1 knockdown partially restored these phosphorylation ratios and endothelial marker expression. Although GSVA reflects relative transcriptional gene-set activity and Western blotting measures protein phosphorylation, the consistent direction of these findings supports an association between the FN1-high remodeling state and impaired PI3K/AKT-related signaling. Nevertheless, the current data do not establish FN1 as a direct upstream regulator of the PI3K/AKT/eNOS pathway. BPA may also affect this signaling axis through oxidative stress, membrane receptors, mitochondrial dysfunction, or other mechanisms. The findings are therefore more consistent with an interconnected process in which FN1-associated extracellular matrix remodeling and reduced endothelial-protective signaling jointly contribute to BPA-induced endothelial abnormalities.

Baicalin is a flavonoid glycoside derived from *Scutellaria baicalensis* and has been reported to reduce oxidative stress and inflammation, inhibit excessive extracellular matrix deposition, and improve endothelial function in several vascular injury models (36–38). Its protective effects may involve PI3K/AKT/eNOS, NF-κB, Nrf2, and TGF-β-related signaling, although the predominant mechanism is likely to depend on the experimental context (36–39). In contrast to conventional pretreatment designs, baicalin was added after 12 h of BPA exposure while BPA remained present. This delayed-intervention design more closely reflects a rescue strategy under continuing exposure conditions. Baicalin improved cell viability, increased PI3K/AKT/eNOS phosphorylation, restored CD31 and VE-cadherin expression, and reduced N-cadherin and vimentin expression. The additional improvement observed in the FN1-knockdown plus baicalin group suggests that the protective effects of baicalin are unlikely to depend entirely on FN1. Antioxidant, anti-inflammatory, mitochondrial-protective, and other extracellular matrix-related mechanisms may also contribute.

Molecular docking and 100-ns MD simulations suggested potential structural compatibility of both BPA and baicalin with the available FN1 domain. Under the present computational conditions, the estimated MM/GBSA binding free energy of the baicalin–FN1 complex was more negative than that of the BPA–FN1 complex. These findings indicate predicted local structural compatibility between the ligands and the isolated FN1 domain under the present computational conditions. Because the simulations were performed using an isolated FN1 domain rather than experimentally determined full-length FN1, the predicted poses reflect only local structural compatibility with the 2EC3 fragment. They do not establish that the identified region represents a physiologically relevant ligand-binding pocket or reproduce the accessibility and conformational environment of full-length FN1 *in vivo*. Moreover, docking, MD simulations, and MM/GBSA calculations without entropic correction do not demonstrate direct binding and cannot establish that baicalin acts by competing with BPA for FN1. Direct interaction and binding specificity will require validation using biophysical approaches such as surface plasmon resonance, microscale thermophoresis, or isothermal titration calorimetry.

A strength of this study was the integration of self-generated DMED corpus cavernosum transcriptomic data, public transcriptomic datasets, human single-cell and spatial transcriptomic data, molecular simulations, and experiments in corpus cavernosum endothelial cells. FN1 knockdown and delayed baicalin intervention also provided preliminary functional evidence. Several limitations should nevertheless be acknowledged. First, candidate-target prioritization partly depended on databases and computational algorithms and may have been influenced by database coverage, data quality, and parameter selection. Second, the study did not include an HG+BPA group or a BPA-exposed DMED animal model; therefore, it cannot determine whether BPA interacts synergistically with high glucose or aggravates established DMED. Third, FN1 validation was mainly based on siRNA-mediated knockdown, whereas FN1 overexpression, rescue experiments, integrin-blocking studies, and cell- or tissue-specific genetic interventions were not performed. Therefore, the present data support a contributory role for FN1 but do not establish that FN1 is sufficient to induce the observed endothelial abnormalities or that the effects of FN1 are specifically mediated through ECM–integrin signaling. In addition, the upstream mechanisms responsible for BPA-induced FN1 upregulation were not investigated. In particular, estrogen receptor- or G protein-coupled estrogen receptor-mediated signaling was not examined using receptor-specific antagonists or related mechanistic interventions. Therefore, the present study cannot determine whether FN1 upregulation occurs downstream of canonical or membrane-associated estrogen-receptor pathways. Receptor-antagonist experiments, temporal analyses, and promoter- or transcription-factor-level studies will be required to clarify the regulation of FN1 in BPA-exposed cavernous endothelial cells. Fourth, the spatial transcriptomic data were obtained from a single normal human corpus cavernosum sample and were used only as an anatomical reference, precluding inference of DMED-specific spatial changes. Fifth, the predicted BPA–FN1 and baicalin–FN1 interactions were not validated using direct binding assays. Sixth, although the BPA concentration range was selected with reference to a previous endothelial-cell study and preliminary CCK-8 assays showed that cell viability remained above 75% after exposure to 150 μM BPA for 24 h, the selected concentration still represented a relatively high, short-term *in vitro* exposure and may not reflect chronic low-dose human exposure. Therefore, broad cellular stress and non-specific cytotoxic effects cannot be completely excluded. Future studies using prolonged or repeated exposure at lower BPA concentrations are needed to determine whether the FN1-associated phenotype persists under conditions more closely approximating human environmental exposure. In addition, dual-marker immunofluorescence, endothelial barrier integrity, collagen secretion, cell migration, tube formation, NO production, cGMP levels, cavernous relaxation, and whole-organ erectile function were not directly evaluated. Accordingly, the observed molecular changes should be interpreted as EndMT-like phenotypic remodeling rather than definitive evidence of complete EndMT. Finally, the relationships among BPA exposure, FN1-associated endothelial remodeling, and erectile dysfunction require confirmation in human populations. Future studies combining chronic low-dose BPA exposure, DMED animal models, FN1-specific genetic manipulation, direct binding assays, and functional erectile assessments are needed to clarify the relevance of FN1-associated endothelial remodeling to the potential effects of BPA on erectile function.

## 5 Conclusion

This study integrated network toxicology, transcriptomic analyses, machine-learning-assisted feature selection, single-cell and spatial transcriptomics, molecular simulations, and *in vitro* experiments to investigate the potential endothelial mechanisms underlying BPA-related erectile dysfunction. FN1 was prioritized as a candidate molecule associated with DMED-relevant cavernous endothelial remodeling. In corpus cavernosum endothelial cells, BPA exposure was accompanied by increased FN1 expression, EndMT-like phenotypic alterations, and reduced PI3K/AKT/eNOS signaling activity. FN1 knockdown and delayed baicalin intervention partially alleviated these abnormalities and improved cell viability. These findings suggest that FN1-associated endothelial remodeling may participate in BPA-related cavernous endothelial injury, while baicalin may exert partial protective effects. Further animal studies, direct binding assays, and clinical investigations are required to clarify the mechanistic relevance and translational significance of these findings.

## Data Availability

The public datasets analyzed in this study are available from the National Center for Biotechnology Information Gene Expression Omnibus database (https://www.ncbi.nlm.nih.gov/geo/) under accession numbers GSE2457, GSE206528, and GSE261085. The self-generated rat corpus cavernosum transcriptomic dataset generated in this study is associated with NCBI BioProject accession number PRJNA1503169. The processed expression matrix, sample metadata, and corresponding analysis code, including the machine-learning-assisted feature-selection pipeline, are available from the corresponding author upon reasonable request.

## Supplementary material

Supplementary Table S1 EndMT-related genes used for module-score analysis.

## References

[1] Vandenberg LN Hauser R Marcus M Olea N Welshons WV. Human exposure to bisphenol A (BPA). Reprod Toxicol. (2007) 24:139–77. doi: 10.1016/j.reprotox.2007.07.010 17825522

[2] Geens T Aerts D Berthot C Bourguignon JP Goeyens L Lecomte P . A review of dietary and non-dietary exposure to bisphenol-A. Food Chem Toxicol. (2012) 50:3725–40. doi: 10.1016/j.fct.2012.07.059 22889897

[3] Rochester JR. Bisphenol A and human health: a review of the literature. Reprod Toxicol. (2013) 42:132–55. doi: 10.1016/j.reprotox.2013.08.008 23994667

[4] Peretz J Vrooman L Ricke WA Hunt PA Ehrlich S Hauser R . Bisphenol a and reproductive health: update of experimental and human evidence, 2007-2013. Environ Health Perspect. (2014) 122:775–86. doi: 10.1289/ehp.1307728 24896072 PMC4123031

[5] Li DK Zhou Z Miao M He Y Qing D Wu T . Relationship between urine bisphenol-A level and declining male sexual function. J Androl. (2010) 31:500–6. doi: 10.2164/jandrol.110.010413 20467048

[6] Li D Zhou Z Qing D He Y Wu T Miao M . Occupational exposure to bisphenol-A (BPA) and the risk of self-reported male sexual dysfunction. Hum Reprod. (2010) 25:519–27. doi: 10.1093/humrep/dep381 19906654

[7] Kovanecz I Gelfand R Masouminia M Gharib S Segura D Vernet D . Chronic high dose intraperitoneal bisphenol A (BPA) induces substantial histological and gene expression alterations in rat penile tissue without impairing erectile function. J Sex Med. (2013) 10:2952–66. doi: 10.1111/jsm.12336 24134786 PMC4038545

[8] Akintunde JK Akintola TE Aliu FH Fajoye MO Adimchi SO. Naringin regulates erectile dysfunction by abolition of apoptosis and inflammation through NOS/cGMP/PKG signalling pathway on exposure to Bisphenol-A in hypertensive rat model. Reprod Toxicol. (2020) 95:123–36. doi: 10.1016/j.reprotox.2020.05.007 32428650

[9] Yafi FA Jenkins L Albersen M Corona G Isidori AM Goldfarb S . Erectile dysfunction. Nat Rev Dis Primers. (2016) 2:16003. doi: 10.1038/nrdp.2016.3 27188339 PMC5027992

[10] Musicki B Burnett AL. Endothelial dysfunction in diabetic erectile dysfunction. Int J Impot Res. (2007) 19:129–38. doi: 10.1038/sj.ijir.3901494 16775612

[11] Hurt KJ Musicki B Palese MA Crone JK Becker RE Moriarity JL . Akt-dependent phosphorylation of endothelial nitric-oxide synthase mediates penile erection. Proc Natl Acad Sci USA. (2002) 99:4061–6. doi: 10.1073/pnas.052712499 11904450 PMC122648

[12] Xiao M Tan X Zeng H Liu B Tang X Xu Y . Yes-associated protein promotes endothelial-mesenchymal transition to mediate diabetes mellitus erectile dysfunction by phosphorylating Smad3. World J Mens Health. (2025) 43:686–701. doi: 10.5534/wjmh.240126 39536767 PMC12257315

[13] Wang E Wang H Chakrabarti S. Endothelial-to-mesenchymal transition: an underappreciated mediator of diabetic complications. Front Endocrinol. (2023) 14:1050540. doi: 10.3389/fendo.2023.1050540 36777351 PMC9911675

[14] Xia M Yuan Y Fang D Tan X Zhao F Li X . Blocking TSP1 ameliorates diabetes mellitus-induced erectile dysfunction by inhibiting the TGF-beta/SMAD pathway. World J Mens Health. (2025) 43:580–94. doi: 10.5534/wjmh.240065 39344111 PMC12257313

[15] He QJ Yin YC Xu YY Tan X Wang YC Zhang W. Mechanistic insights into bisphenol A - induced liver fibrosis: evidence of PPARgamma downregulation and AKT1/FN1 signaling from multi-level analysis. Ecotoxicol Environ Saf . (2025) 303:119068. doi: 10.1016/j.ecoenv.2025.119068 40961596

[16] Yang M Mao K Cao X Liu H Mao W Hao L. Integrated network toxicology, transcriptomics and gut microbiomics reveals hepatotoxicity mechanism induced by benzo[a]pyrene exposure in mice. Toxicol Appl Pharmacol. (2024) 491:117050. doi: 10.1016/j.taap.2024.117050 39111554

[17] Sturla SJ Boobis AR FitzGerald RE Hoeng J Kavlock RJ Schirmer K . Systems toxicology: from basic research to risk assessment. Chem Res Toxicol. (2014) 27:314–29. doi: 10.1021/tx400410s 24446777 PMC3964730

[18] Pudjihartono N Fadason T Kempa-Liehr AW O'Sullivan JM. A review of feature selection methods for machine learning-based disease risk prediction. Front Bioinform. (2022) 2:927312. doi: 10.3389/fbinf.2022.927312 36304293 PMC9580915

[19] Cheng Y Xu SM Santucci K Lindner G Janitz M. Machine learning and related approaches in transcriptomics. Biochem Biophys Res Commun. (2024) 724:150225. doi: 10.1016/j.bbrc.2024.150225 38852503

[20] Park J Kim J Lewy T Rice CM Elemento O Rendeiro AF . Spatial omics technologies at multimodal and single cell/subcellular level. Genome Biol. (2022) 23:256. doi: 10.1186/s13059-022-02824-6 36514162 PMC9746133

[21] Kulkarni A Anderson AG Merullo DP Konopka G. Beyond bulk: a review of single cell transcriptomics methodologies and applications. Curr Opin Biotechnol. (2019) 58:129–36. doi: 10.1016/j.copbio.2019.03.001 30978643 PMC6710112

[22] Meng C Jiang J Jiang R. Upregulated FTO promotes TNIP1 m6A demethylation induced penile corpus cavernosum endothelial pyroptosis and erectile dysfunction in type 2 diabetic rats. J Sex Med. (2026) 23:qdag119. doi: 10.1093/jsxmed/qdag119 42015403

[23] Wu T Hu E Xu S Chen M Guo P Dai Z . clusterProfiler 40: a universal enrichment tool for interpreting omics data. Innovation. (2021) 2:100141. doi: 10.1016/j.xinn.2021.100141 34557778 PMC8454663

[24] Kanehisa M Furumichi M Sato Y Kawashima M Ishiguro-Watanabe M. KEGG for taxonomy-based analysis of pathways and genomes. Nucleic Acids Res. (2023) 51:D587–92. doi: 10.1093/nar/gkac963 36300620 PMC9825424

[25] Gene Ontology Consortium Aleksander SA Balhoff J Carbon S Cherry JM Drabkin HJ. The Gene Ontology knowledgebase in 2023. Genetics. (2023) 224:iyad031. doi: 10.1093/genetics/iyad031 36866529 PMC10158837

[26] Mohsenzadeh MS Razavi BM Imenshahidi M Mohajeri SA Rameshrad M Hosseinzadeh H. Evaluation of green tea extract and epigallocatechin gallate effects on bisphenol A-induced vascular toxicity in isolated rat aorta and cytotoxicity in human umbilical vein endothelial cells. Phytother Res. (2021) 35:996–1009. doi: 10.1002/ptr.6861 32893422

[27] Rochester JR Bolden AL. Bisphenol S and F: a systematic review and comparison of the hormonal activity of bisphenol A substitutes. Environ Health Perspect. (2015) 123:643–50. doi: 10.1289/ehp.1408989 25775505 PMC4492270

[28] Moon MK Jeong IK Jung Oh T Ahn HY Kim HH Park YJ . Long-term oral exposure to bisphenol A induces glucose intolerance and insulin resistance. J Endocrinol. (2015) 226:35–42. doi: 10.1530/JOE-14-0714 25972359

[29] Feng C Yang Y Chen L Guo R Liu H Li C . Prevalence and characteristics of erectile dysfunction in obstructive sleep apnea patients. Front Endocrinol. (2022) 13:812974. doi: 10.3389/fendo.2022.812974 35250871 PMC8896119

[30] Pankov R Yamada KM. Fibronectin at a glance. J Cell Sci. (2002) 115(Pt 20):3861–3. doi: 10.1242/jcs.00059 12244123

[31] Dalton CJ Lemmon CA. Fibronectin: molecular structure, fibrillar structure and mechanochemical signaling. Cells. (2021) 10:2443. doi: 10.20944/preprints202108.0010.v1 34572092 PMC8471655

[32] Bonnans C Chou J Werb Z. Remodelling the extracellular matrix in development and disease. Nat Rev Mol Cell Biol. (2014) 15:786–801. doi: 10.1038/nrm3904 25415508 PMC4316204

[33] Huang Q Sheibani N. High glucose promotes retinal endothelial cell migration through activation of Src, PI3K/Akt1/eNOS, and ERKs. Am J Physiol Cell Physiol. (2008) 295:C1647–57. doi: 10.1152/ajpcell.00322.2008 18945941 PMC2603562

[34] Kovacic JC Dimmeler S Harvey RP Finkel T Aikawa E Krenning G . Endothelial to mesenchymal transition in cardiovascular disease: JACC state-of-the-art review. J Am Coll Cardiol. (2019) 73:190–209. doi: 10.1016/j.jacc.2018.09.089 30654892 PMC6865825

[35] Musicki B Kramer MF Becker RE Burnett AL. Inactivation of phosphorylated endothelial nitric oxide synthase (Ser-1177) by O-GlcNAc in diabetes-associated erectile dysfunction. Proc Natl Acad Sci USA. (2005) 102:11870–5. doi: 10.1073/pnas.0502488102 16085713 PMC1187969

[36] Wei X Zhu X Hu N Zhang X Sun T Xu J . Baicalin attenuates angiotensin II-induced endothelial dysfunction. Biochem Biophys Res Commun. (2015) 465:101–7. doi: 10.1016/j.bbrc.2015.07.138 26239661

[37] Si L Lai Y. Pharmacological mechanisms by which baicalin ameliorates cardiovascular disease. Front Pharmacol. (2024) 15:1415971. doi: 10.3389/fphar.2024.1415971 39185317 PMC11341428

[38] Hu Q Gao L Peng B Liu X. Baicalin and baicalein attenuate renal fibrosis *in vitro* via inhibition of the TGF-beta1 signaling pathway. Exp Ther Med. (2017) 14:3074–80. doi: 10.3892/etm.2017.4888 28928802 PMC5590043

[39] Hou Y Liang Z Qi L Tang C Liu X Tang J . Baicalin targets HSP70/90 to regulate PKR/PI3K/AKT/eNOS signaling pathways. Molecules. (2022) 27:1432. doi: 10.3390/molecules27041432 35209223 PMC8874410

